# A novel risk model of three gefitinib-related genes FBP1, SBK1 and AURKA is related to the immune microenvironment and is predicting prognosis of lung adenocarcinoma patients

**DOI:** 10.18632/aging.205040

**Published:** 2023-09-21

**Authors:** Qiang Guo, Kai Li, Ni Jiang, Rui Zhou, Xin-Rui Rao, Chuang-Yan Wu

**Affiliations:** 1 Department of Thoracic Surgery, Union Hospital, Tongji Medical College, Huazhong University of Science and Technology, Wuhan, China; 2 Department of Cardiothoracic Surgery, Taihe Hospital, Hubei University of Medicine, Shiyan, China; 3 Department of Hepatobiliary and Pancreatic Surgery, The People’s Hospital of Jianyang City, Jianyang, China; 4 Department of Obstetrics and Gynecology, Women and Children’s Hospital of Chongqing Medical University, Chongqing, China; 5 Cancer Center, Union Hospital, Tongji Medical College, Huazhong University of Science and Technology, Wuhan, China; 6 Institute of Radiation Oncology, Union Hospital, Tongji Medical College, Huazhong University of Science and Technology, Wuhan, China

**Keywords:** Gefitinib, FBP1, SBK1, AURKA, LUAD, risk model, nomogram

## Abstract

Purpose: Gefitinib, an anticancer drug, has been reported to potentially improve the prognosis of patients with lung adenocarcinoma (LUAD). This study aims to investigate the roles and mechanisms of Gefitinib.

Methods: The effects of Gefitinib on the growth and migration of LUAD cells were assessed using various methods, including CCK-8, flow cytometry, wound healing, and Transwell assays. To analyze the function and mechanisms of the differentially expressed Gefitinib target genes (GTGs), data from the TCGA database were utilized. Kaplan-Meier survival and ROC analysis identified prognostic-related GTGs and constructed a prognostic nomogram in LUAD. Consensus clustering, COX analysis and survival analysis evaluated the relationship between GTGs and the prognosis of LUAD patients. The mechanisms of the risk model involved LUAD progression, and the relationship between the risk model and immune microenvironment were investigated.

Results: Gefitinib could inhibit proliferation, migration and invasion and promote cell apoptosis. 84 DEGTGs were involved in RAS, MAPK, ERBB pathways. The DEGTGs (FBP1, SBK1, and AURKA) were the independent risk factors for dismal prognosis of LUAD patients and were used to establish risk model and nomogram. Gefitinib could promote the expression of FBP1 and inhibit the expression of SBK1 and AURKA. High-risk LUAD patients had the dismal prognosis, and the high-risk score group was significantly associated with the immune microenvironment.

Conclusion: FBP1, SBK1, and AURKA are prognostic risk factors, and the risk model and nomogram of FBP1, SBK1 and AURKA are associated with dismal prognosis and immune cell infiltration, and have huge prospects for application in evaluating the prognosis in LUAD.

## INTRODUCTION

Lung adenocarcinoma (LUAD) is widely acknowledged as a common subtype of lung cancer [1, 2]. Current evidence suggests that the incidence of LUAD has increased in recent years. However, dismal survival rates have been reported for LUAD patients. Overwhelming evidence substantiates that some biomarkers can be used to predict the prognosis of LUAD patients. Interestingly, inhibiting or promoting the expression of these biomarkers could delay LUAD progression [3–5]. For instance, it has been shown that thyroid hormone receptor interactor 13 (TRIP13) is significantly expressed in LUAD tissues, negatively correlated with mortality in LUAD patients, and involved in activating the AKT/mTORC1/c-Myc signaling pathway to promote LUAD cell proliferation, migration and invasion [3]. Moreover, the expression levels of CAPN1, c-MET, and PIK3R2 are reportedly upregulated, while PTPN1 is downregulated in LUAD. CAPN1 could activate the c-Met/PIK3R2 pathway by promoting the degradation of PTPN1 protein to enhance the malignant behavior and erlotinib resistance of LUAD cells [5].

It has been shown that Gefitinib could inhibit the catalytic activity of epidermal growth factor receptor (EGFR), inhibiting tyrosine kinase-dependent tumor growth, cell cycle arrest, and angiogenesis. Gefitinib could compete with the ATP and EGFR tyrosine kinase domains for binding and inhibit receptor autophosphorylation and signaling transduction. An increasing body of evidence suggests that Gefitinib plays an important role against LUAD progression [6–9]. For instance, a study showed that among 121 patients with EGFR-mutant advanced LUAD, chemotherapy plus Gefitinib yielded significantly higher progression-free survival (PFS) and overall survival (OS) than chemotherapy or Gefitinib. The overall response rate (ORR) was significantly better in the chemotherapy plus Gefitinib groups than in the chemotherapy or Gefitinib groups. Chemotherapy combined with the Gefitinib therapy could provide a better survival benefit to patients with LUAD with sensitive EGFR mutation [6]. In EGFR TKI-sensitive cells PC-9, the expression of LPCAT1 was significantly increased in EGFR TKI-resistant cells PC-9R. Interestingly, the formation of the LPCAT1-EGFR positive feedback loop could regulate the EGFR/PI3K/AKT signaling pathway to promote Gefitinib resistance to LUAD [8]. Lysine demethylase 5A (KDM5A) is a histone demethylase that is upregulated in LUAD tissues and cells. It has been shown that interfering with KDM5A expression led to apoptosis, inhibited cell proliferation, and promoted the sensitivity of cancer cells to Gefitinib, accompanied by upregulation of BAX protein expression and downregulation of Bcl-2 protein expression in LUAD. Overexpression of KDM5A could promote the proliferation of LUAD cells, inhibit apoptosis, and promote the resistance of cancer cells to Gefitinib [9]. Although Gefitinib treatment could improve the prognosis and delay cell progression in LUAD, the roles of Gefitinib hub target genes in LUAD progression remain poorly understood. Therefore, the Gefitinib hub target molecules in the progression of LUAD were identified, and a risk model of Gefitinib target genes was constructed to predict the prognosis of LUAD patients using bioinformatics and cell experiments.

## RESULTS

### Gefitinib could inhibit cell growth and migration in LUAD

**Figure 1:**
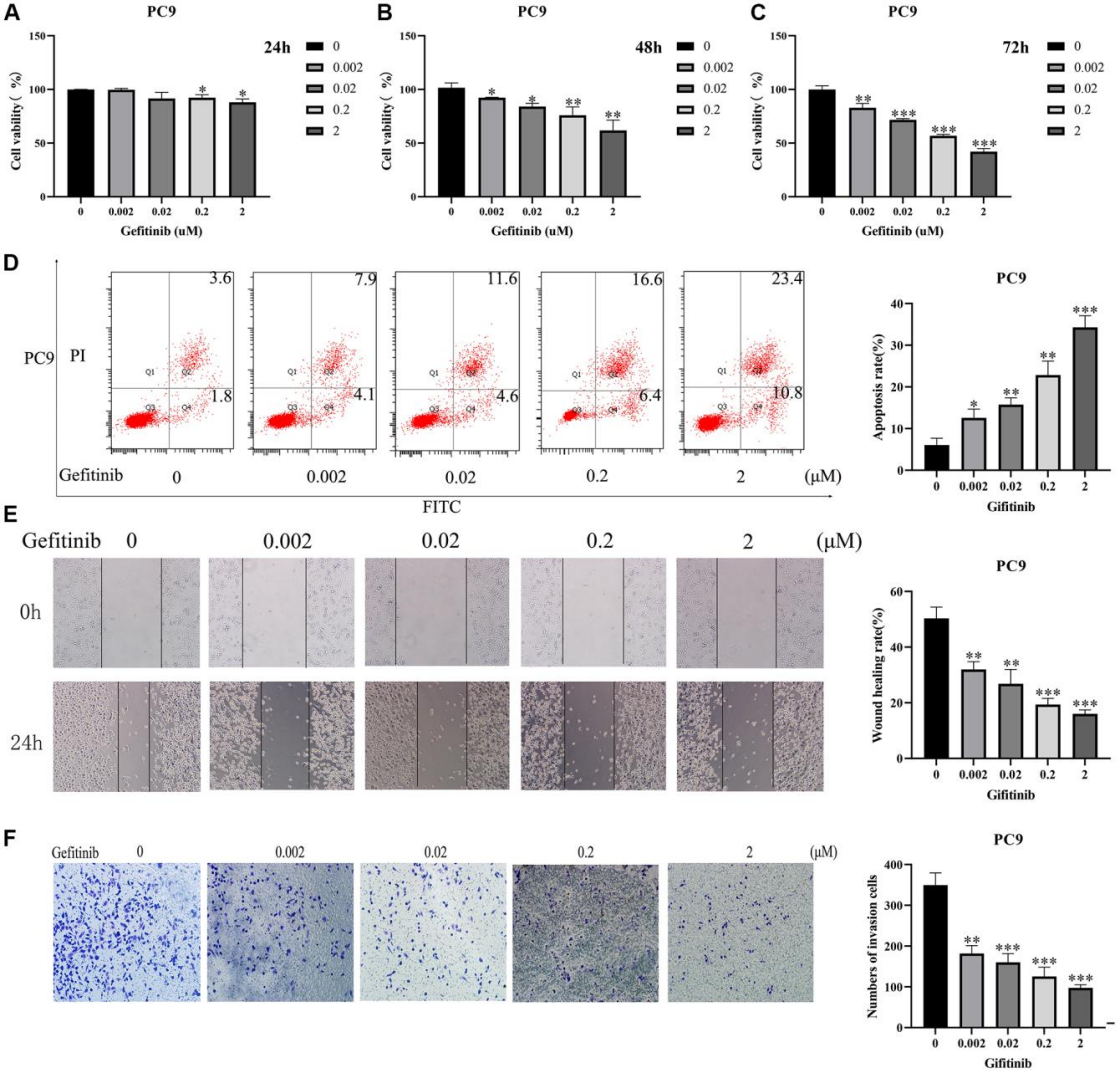
**Gefitinib could inhibit cancer cell growth and migration.** (**A**–**C**) Cell viability; (**D**) Cell apoptosis; (**E**) Cell Migration; (**F**) Cell Invasion.

After adding an appropriate concentration of Gefitinib, we found that with increased Gefitinib concentration, the proliferative ability of cells decreased during the CCK-8 assay (Figure 1A–1C), and the apoptosis rate significantly raised during flow cytometry (Figure 1D). In addition, Gefitinib significantly inhibited cell migration and invasion during wound healing and Transwell assays (Figure 1E, 1F).

### The expression levels of Gefitinib target genes in LUAD

**Figure 2:**
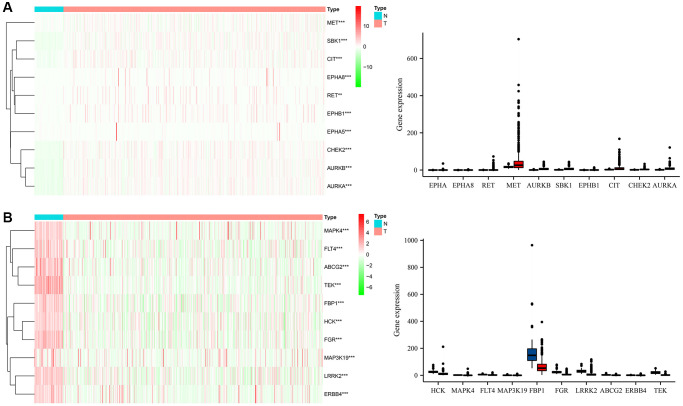
**The expression levels of Gefitinib target genes in LUAD tissues.** (**A**) High expression; (**B**) Low expression. Abbreviations: LUAD: Lung adenocarcinoma; T: LUAD tissues; N: normal tissues.

Gefitinib targets approximately 100 genes, primarily kinases (Table 1). Compared with normal lung tissues, 84 DEGs were identified in LUAD tissues (Table 2). The top 10 DEGs were visualized in a heatmap and histogram based on the fold change (Figure 2).

**Table 1:** Gefitinib target genes.

**Target genes**			
HIPK4	IRAK4	RIPK2	FGFR1
SBK1	PRKD1	ULK3	FBP1
PIP4K2C	LYN	EPHB1	BRAF
ERBB2	STK17B	IRAK3	AURKA
ABL1	STK10	MAPK6	FLT4
KIT	EPHA5	EPHB4	LRRK2
FLT3	PHKG1	DMPK	PDGFRB
EGFR	ABL2	NLK	AURKB
RET	EPHA8	ABCG2	TEK
BLK	MAPK9	PIM3	CLK1
PHKG2	SLK	MINK1	TGFBR1
DAPK3	MKNK2	CIT	MAP2K1
CHEK2	FRK	LTK	JAK3
LCK	STK36	NUAK2	DDR1
MAPK10	GAK	SIK2	COQ8A
SRC	TXK	MAPK4	INSR
MYLK2	FGR	EPHA1	TNNI3K
KDR	STK17A	EPHB6	MAPK14
CSNK1D	EPHA6	ERBB3	DYRK1A
ERBB4	TNIK	MAP3K2	PDGFRA
CDK7	MKNK1	MAP3K3	HUNK
RPS6KA4	AXL	DCLK3	FYN
HCK	CSNK1E	MAP3K19	YES1
IRAK1	MAP2K5	FLT1	RPS6KA1
MET	EPHA3	EPHA2	CSK

**Table 2:** 84 DEGs in LUAD tissues.

**Gene**	**logFC**	***P***	**Gene**	**logFC**	***P***
SIK2	−0.823022668	1.27E-19	EPHA1	1.021486338	2.65E-14
PRKD1	−0.815043872	7.85E-16	TXK	−0.199840205	6.13E-07
MKNK2	−0.437153109	4.11E-11	EPHB4	0.424531032	0.000179901
CSK	0.234775877	0.00016932	DAPK3	−0.191517716	0.006880896
FRK	1.104629696	3.57E-16	DCLK3	0.619636828	0.00052685
HIPK4	−0.450786343	1.84E-06	STK17B	−0.220423503	1.61E-05
ULK3	0.713039467	6.39E-15	PIM3	0.31973622	0.026381134
HCK	−1.165212956	1.95E-23	SBK1	2.687143232	4.93E-21
EPHA5	2.345418573	6.18E-07	AXL	−0.849954085	2.15E-20
HUNK	0.09674773	0.030787751	STK10	−0.636132433	8.03E-15
MAPK6	0.725789271	2.39E-12	LRRK2	−1.83742934	1.99E-26
FLT3	−0.1398755	0.00299809	EPHB1	1.659989452	0.000183369
EPHA8	4.28048008	1.29E-10	CIT	2.153606791	1.55E-10
RET	3.069514219	0.008169043	CHEK2	1.617485001	7.44E-28
TNNI3K	−0.062878035	0.000303105	JAK3	1.263929465	3.54E-18
ERBB2	1.17003348	7.02E-16	FLT1	−0.487731672	1.72E-06
GAK	0.456027428	1.55E-07	PDGFRB	−0.084970938	0.043391534
EPHB6	−0.725722081	6.65E-20	MAP3K3	−0.883369078	7.21E-27
MAPK4	−1.381170977	6.74E-26	MAPK14	0.221450042	0.000785059
PIP4K2C	0.769030841	2.34E-17	RPS6KA1	−0.437722081	2.89E-12
FLT4	−1.224022669	1.75E-20	RPS6KA4	0.475941746	5.32E-09
MAPK10	−0.858238831	5.28E-18	ABCG2	−1.89336698	5.70E-30
ABL1	0.147380872	0.049905786	STK36	0.772784045	8.06E-11
MAP3K19	−1.413469509	1.19E-07	NUAK2	0.816006757	2.61E-10
IRAK3	−0.501638979	1.69E-12	ERBB4	−1.508245411	3.70E-25
MET	1.424801167	0.000129599	FGFR1	−0.16161865	1.21E-08
IRAK1	1.354218442	6.37E-30	MAP2K5	−0.26725795	3.39E-10
RIPK2	0.4578374	0.000760452	MINK1	−0.448686448	1.12E-11
PDGFRA	−0.195864444	0.000547589	KIT	0.521094359	0.031286428
SLK	−0.148783403	0.000564055	INSR	0.386078443	0.00444343
LYN	0.428904666	1.71E-05	CSNK1D	0.394292094	6.97E-12
FBP1	−1.503007393	4.34E-25	CSNK1E	0.505317592	7.12E-09
ABL2	0.480008559	1.26E-06	SRC	0.681725458	4.50E-13
IRAK4	0.405731499	5.13E-08	CDK7	0.727250182	1.99E-21
PHKG2	0.969237137	1.19E-24	FYN	−0.47858826	8.94E-09
DDR1	0.937520074	1.48E-17	KDR	−0.522808212	1.90E-10
MAP3K2	0.30325161	0.010702075	AURKA	2.774021963	2.56E-31
FGR	−1.931309907	5.10E-32	MYLK2	0.958683785	4.57E-08
BLK	0.843421358	2.33E-05	TEK	−3.092499222	3.22E-35
AURKB	3.590111346	8.17E-33	ERBB3	0.956775604	6.37E-13
MKNK1	−0.094286595	0.041519321	DYRK1A	−0.03193067	0.030787751
TNIK	−0.287891618	4.90E-07	NLK	0.312268002	0.00010209

Abbreviations: LUAD: lung adenocarcinoma; DEGs: differentially expressed genes; FC: fold change.

### Biological functions, signaling mechanisms and protein-protein interaction (PPI) networks of the differentially expressed Gefitinib target genes (DEGTGs)

**Figure 3:**
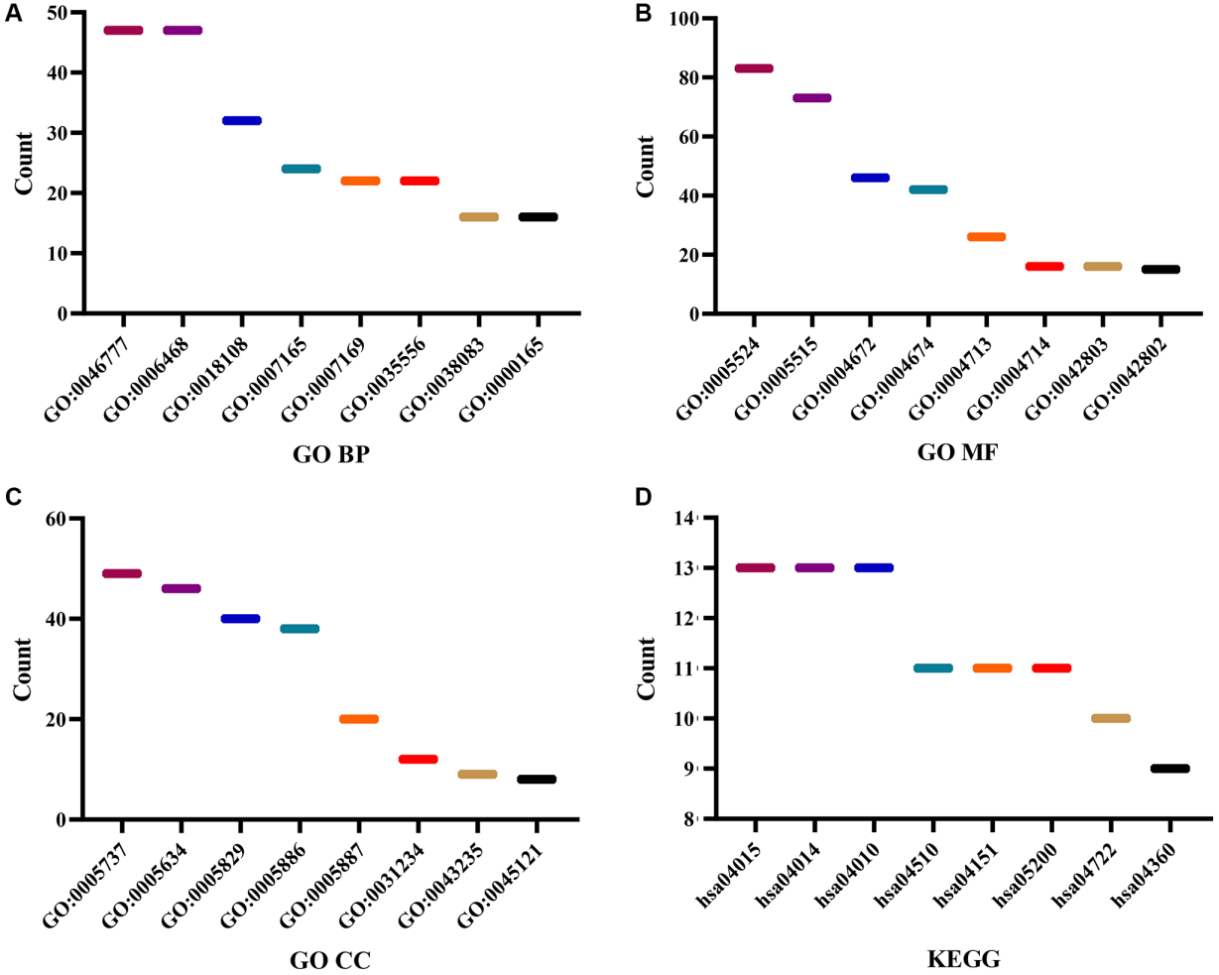
**Functions and mechanisms of the DEGTGs.** (**A**) BP; (**B**) MF; (**C**) CC; (**D**) KEGG. Abbreviations: DEGTGs: differentially expressed Gefitinib target genes; GO: gene ontology; KEGG: Kyoto Encyclopedia of Genes and Genomes; BP: biological process; MF: molecular function; CC: cell component.

**Figure 4:**
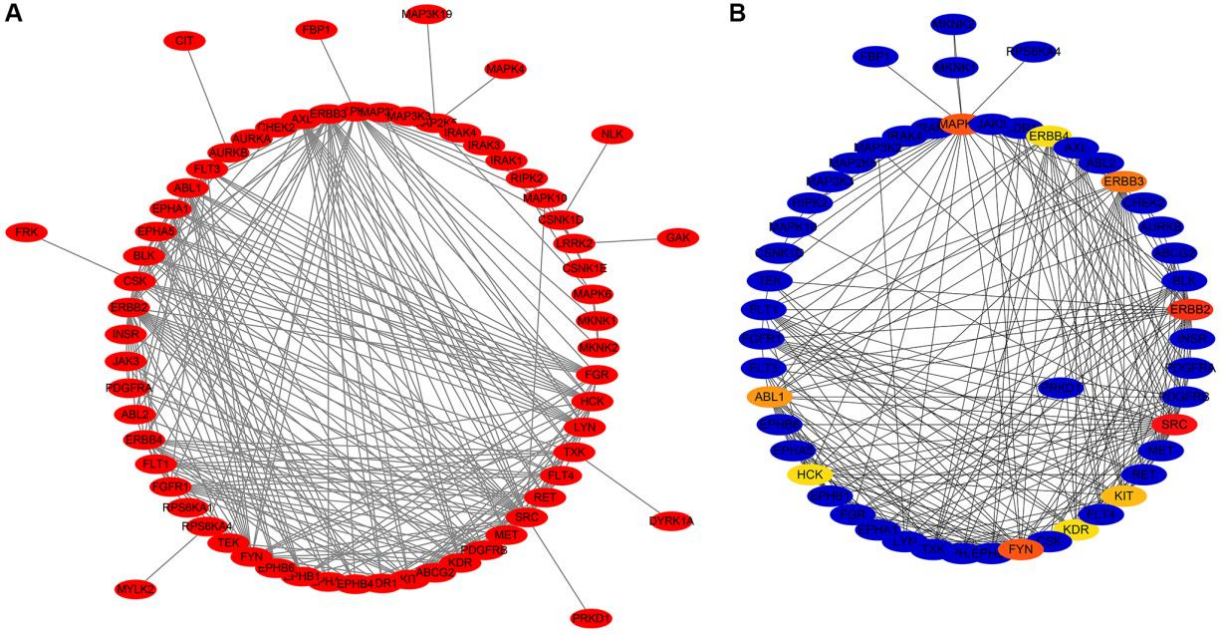
**PPI network of the DEGTGs.** (**A**) PPI network; (**B**) Hub DEGTGs in PPI network. Abbreviations: DEGTGs: differentially expressed Gefitinib target genes; PPI: protein-protein interaction.

Gene ontology (GO) enrichment analysis revealed that the DEGTGs were significantly enriched in the intracellular signaling transduction, positive regulation of phosphatidylinositol 3-kinase activity, positive regulation of phosphatidylinositol 3-kinase signaling, receptor signaling protein tyrosine kinase activity, kinase activity, signal transduction, vascular endothelial growth factor-activated receptor activity, positive regulation of cell migration, vascular endothelial growth factor receptor signaling pathway, receptor complex, positive regulation of mitogen-activated protein kinase (MAPK) cascade, cell migration, growth factor binding, regulation of cell proliferation, negative regulation of apoptotic process, cell cycle, JNK cascade and others (Figure 3A–3C and Supplementary Table 1). Kyoto Encyclopedia of Genes and Genomes (KEGG) pathway analysis revealed that the DEGTGs were enriched in the Rap1, Ras, MAPK, ERBB, PI3K-AKT, HIF-1, pathways in cancer, calcium, insulin, GnRH, and other signaling pathways (Figure 3D and Table 3). A PPI network constructed based on the DEGTGs is shown in Figure 4A, and the key genes in the PPI network are revealed in Figure 4B.

**Table 3:** Signaling pathways of the DEGs in LUAD.

**Term**	**Pathways**	**Count**	***P***
hsa04015	Rap1 signaling pathway	13	1.84E-07
hsa04014	Ras signaling pathway	13	4.10E-07
hsa04722	Neurotrophin signaling pathway	10	7.75E-07
hsa05230	Central carbon metabolism in cancer	8	1.13E-06
hsa04010	MAPK signaling pathway	13	1.37E-06
hsa04510	Focal adhesion	11	9.51E-06
hsa04360	Axon guidance	9	1.24E-05
hsa04520	Adherens junction	7	3.15E-05
hsa04012	ErbB signaling pathway	7	9.96E-05
hsa05120	Epithelial cell signaling in Helicobacter pylori infection	6	2.72E-04
hsa04151	PI3K-Akt signaling pathway	11	7.06E-04
hsa04540	Gap junction	6	9.60E-04
hsa04066	HIF-1 signaling pathway	6	0.001419877
hsa05205	Proteoglycans in cancer	8	0.001653828
hsa05200	Pathways in cancer	11	0.001904711
hsa05131	Shigellosis	5	0.002249456
hsa04020	Calcium signaling pathway	7	0.004459107
hsa04910	Insulin signaling pathway	6	0.006817296
hsa04912	GnRH signaling pathway	5	0.007948726
hsa05152	Tuberculosis	6	0.018599898
hsa04664	Fc epsilon RI signaling pathway	4	0.021171958
hsa05169	Epstein-Barr virus infection	5	0.021410206
hsa05218	Melanoma	4	0.023707687
hsa04611	Platelet activation	5	0.026308393
hsa05133	Pertussis	4	0.027334651
hsa04068	FoxO signaling pathway	5	0.02899109
hsa04810	Regulation of actin cytoskeleton	6	0.035535444
hsa05215	Prostate cancer	4	0.041048942
hsa05219	Bladder cancer	3	0.049367551

Abbreviations: LUAD: lung adenocarcinoma; DEGs: differentially expressed genes.

### Construction of a nomogram based on the DEGTGs

**Figure 5:**
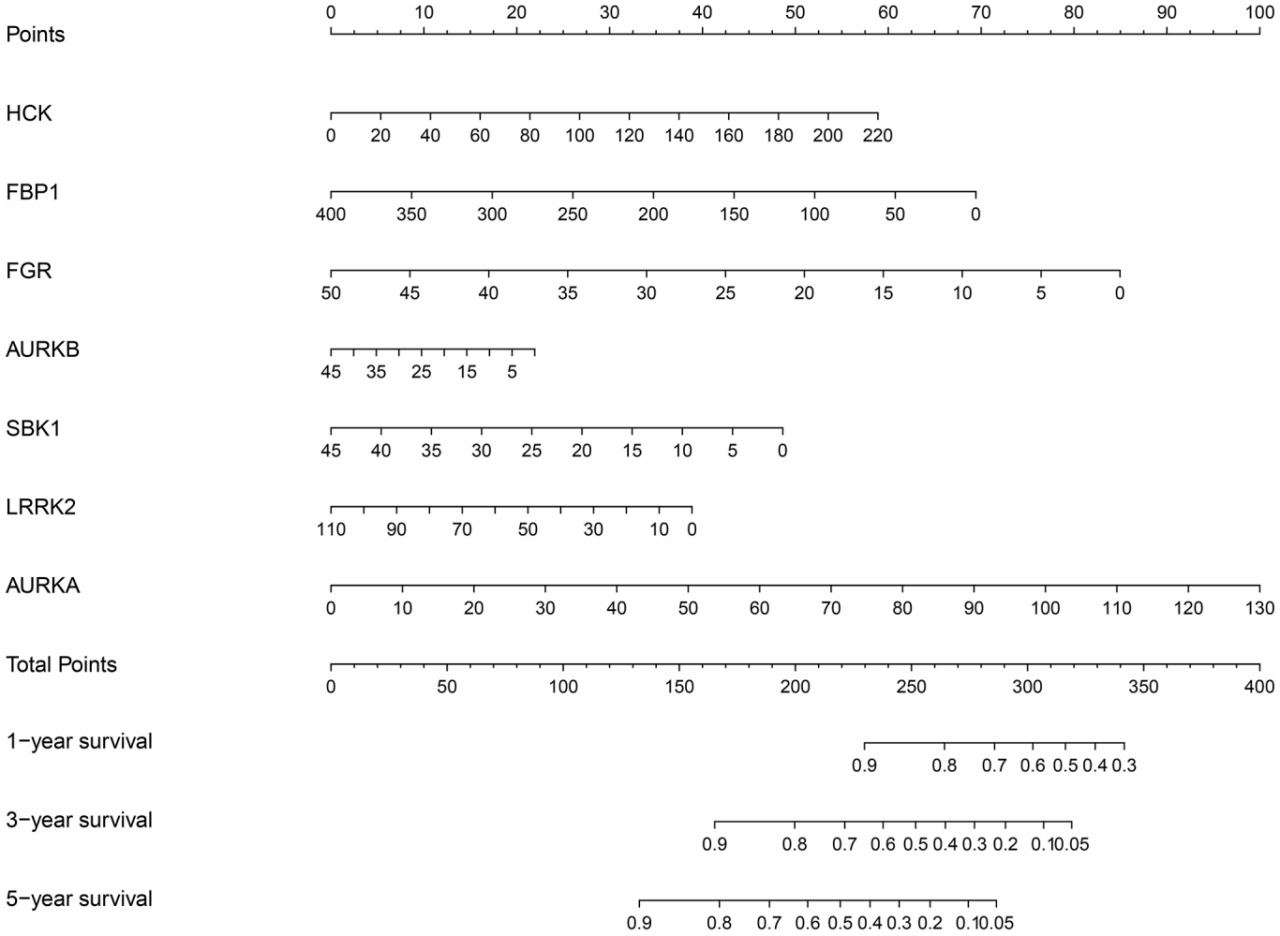
**Nomogram of the DEGTGs in LUAD.** Abbreviations: DEGTGs: differentially expressed Gefitinib target genes; LUAD: lung adenocarcinoma.

Survival analysis revealed that AURKA, AURKB, FBP1, FGR, HCK, LRRK2, and SBK1 levels were associated with poor prognosis in LUAD patients (Table 4). Receiver operating characteristic (ROC) analysis revealed that AURKA, AURKB, FBP1, FGR, HCK, LRRK2, and SBK1 had diagnostic values for LUAD, with the area under the curve (AUCs) of 0.961, 0.973, 0.910, 0.967, 0.895, 0.922, and 0.873, respectively. Based on the results of survival and ROC analysis, we constructed a nomogram based on the AURKA, AURKB, FBP1, FGR, HCK, LRRK2, and SBK1 (Figure 5).

**Table 4:** The prognosis values of the DEGs in LUAD.

**DEGs**	**HR**	**95% CI**	***P***
AURKA	1.51	1.11–2.05	0.009
AURKB	1.48	1.09–2.02	0.013
FBP1	0.57	0.42–0.78	<0.001
FGR	0.63	0.46–0.86	0.003
HCK	0.72	0.53–0.98	0.037
LRRK2	0.73	0.53–0.99	0.044
SBK1	0.67	0.49–0.91	0.01

Abbreviations: LUAD: lung adenocarcinoma; HR: hazard ratio; DEGs: differentially expressed genes; CI: confidence interval.

### Functions and clinical values involved in subgroups of the DEGTGs

**Figure 6:**
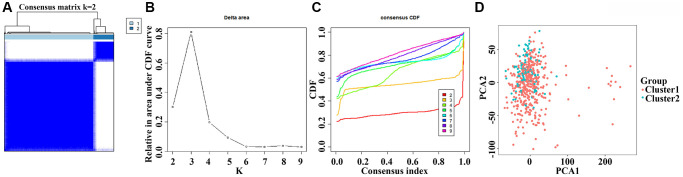
**The clinical roles involved in subgroups of the DEGTGs in LUAD.** Abbreviations: DEGTGs: differentially expressed Gefitinib target genes; LUAD: lung adenocarcinoma.

**Figure 7:**
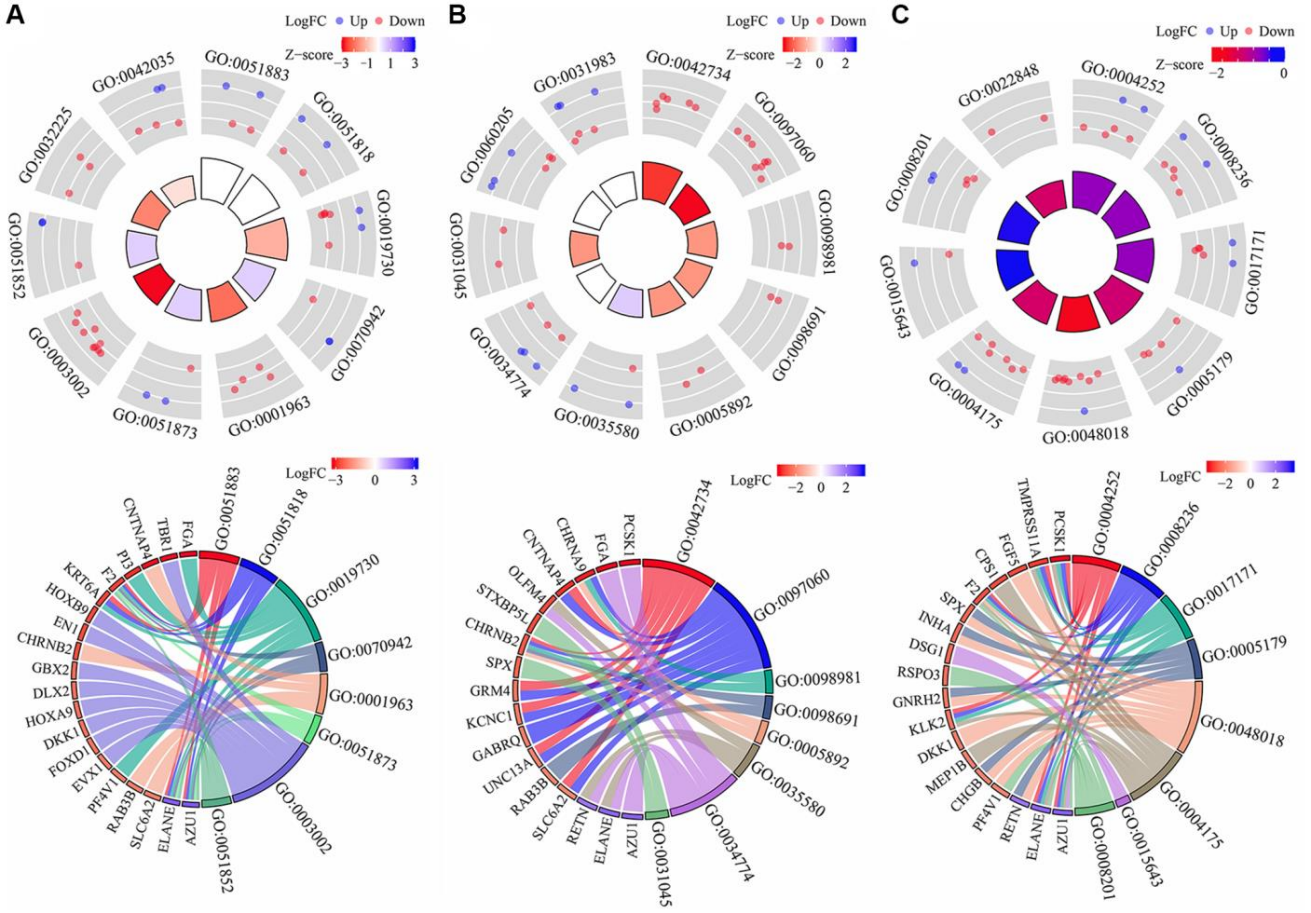
**Functions involved in subgroups of the DEGTGs in LUAD.** (**A**) BP; (**B**) CC; (**C**) MF. Abbreviations: DEGTGs: differentially expressed Gefitinib target genes; GO: gene ontology; KEGG: Kyoto Encyclopedia of Genes and Genomes; BP: biological process; MF: molecular function; CC: cell component; LUAD: lung adenocarcinoma.

**Figure 8:**
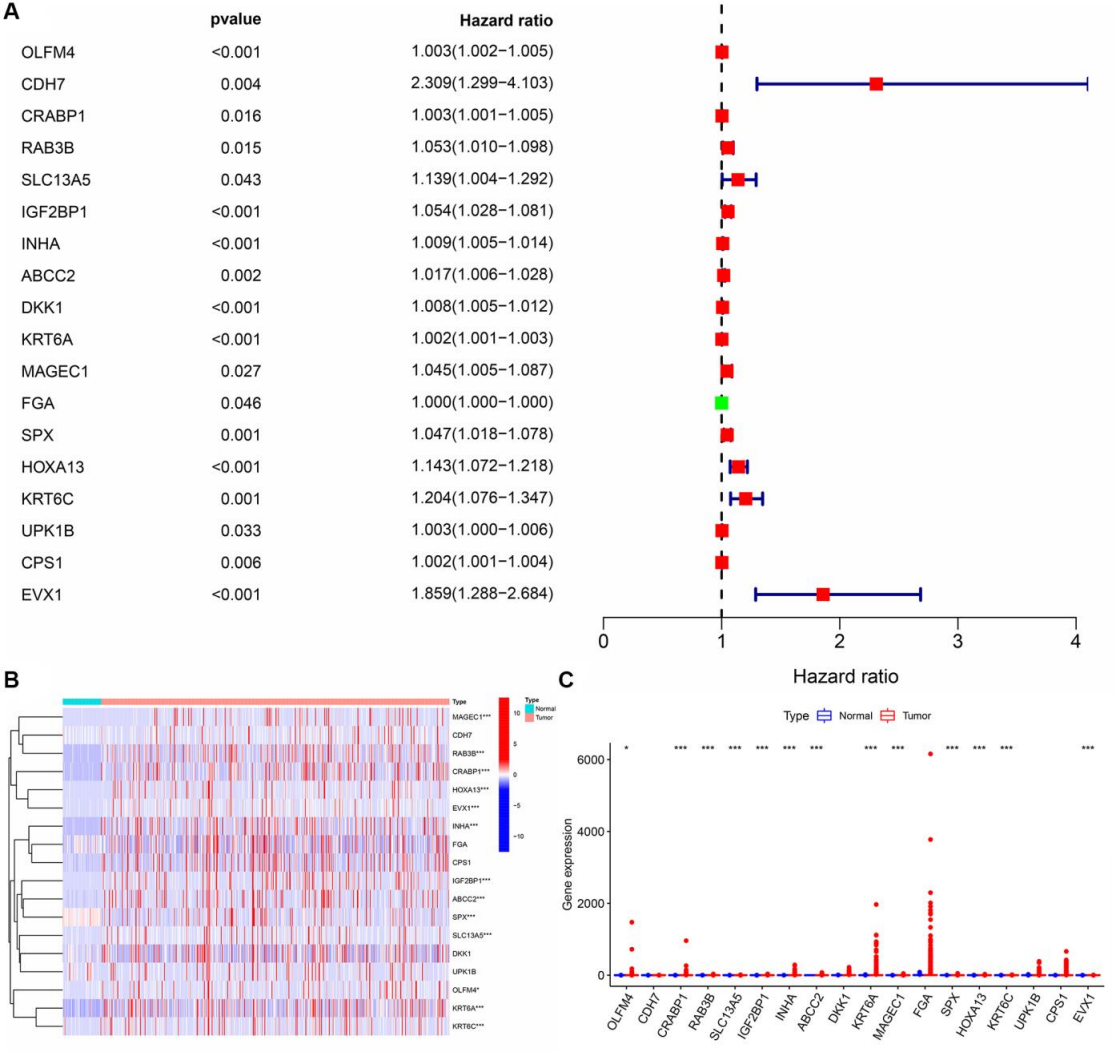
**The prognosis values and levels of the DEGTGs in LUAD.** (**A**) Prognosis related genes; (**B**, **C**) The levels of DEGTGs in LUAD. Abbreviations: DEGTGs: differentially expressed Gefitinib target genes; LUAD: lung adenocarcinoma.

When LUAD patients were classified based on the AURKA, AURKB, FBP1, FGR, HCK, LRRK2, and SBK1 expression levels and K = 2, PCA analysis revealed significant differences between cluster1 and cluster2 (Figure 6). According to our screening criteria analysis, 93 DEGs were found in LUAD tissues of cluster 2 compared with cluster 1 (Table 5). 93 DEGs were significantly enriched in neutrophil-mediated cytotoxicity, regulation of cytokine biosynthetic process, killing of cells of other organisms, disruption of cells of other organisms, regulation of neurotransmitter levels, cytokine biosynthetic process, cytokine metabolic process, positive regulation of response to drug, drug transport, cytolysis, hormone activity and others (Figure 7 and Supplementary Table 2). Cox regression analysis revealed that the expression levels of OLFM4, CDH7, CRABP1, RAB3B, SLC13A5, IGF2BP1, INHA, ABCC2, DKK1, KRT6A, MAGEC1, FGA, SPX, HOXA13, KRT6C, UPK1B, CPS1, and EVX1 were significantly associated with the OS in LUAD (Figure 8A). The levels of OLFM4, CDH7, CRABP1, RAB3B, SLC13A5, IGF2BP1, INHA, ABCC2, DKK1, KRT6A, MAGEC1, FGA, SPX, HOXA13, KRT6C, UPK1B, CPS1, and EVX1 in normal tissues and LUAD tissues are shown in a heatmap and histogram (Figure 8B, 8C).

**Table 5:** The DEGs in the LUAD tissues of clusters 1 and 2.

**Gene**	**logFC**	**DFR**	**Gene**	**logFC**	**DFR**	**Gene**	**logFC**	**DFR**
MYBPH	2.386784135	^*^	MAGEC1	−4.746262844	^*^	EIF4E1B	2.117324727	^*^
CSAG1	−2.121155629	^*^	HIST1H2BB	−2.024017432	^***^	RHCG	−2.887894921	^**^
OLFM4	−2.854804976	^*^	GBX2	−2.270227346	^**^	CEACAM8	2.373919231	^***^
CHRNB2	−2.648571164	^*^	HOXA9	−2.197847501	^*^	TAS2R19	−2.050800865	^***^
F2	−2.77987994	^**^	PCSK1	−3.499863036	^***^	UPK1B	−4.267455858	^*^
FGF5	−2.85157907	^*^	MUC2	−2.721128573	^**^	LVRN	−2.24465015	^*^
CERS3	−3.845929507	^*^	EN1	−2.674544647	^*^	AKR1B10	−2.587706792	^**^
DLX2	−2.204529845	^*^	CPA4	−2.235707486	^**^	PF4V1	−2.078641696	^**^
STXBP5L	−2.843404586	^*^	FGA	−3.320329815	^**^	GNRH2	−2.242805879	^*^
CDH7	−3.039737981	^***^	CYP4F3	−2.409142625	^*^	RSPO3	−2.364153182	^*^
ZMAT4	−2.068479897	^*^	OTOG	−2.091090919	^***^	UNC13A	−2.090243089	^*^
DSG1	−2.450943891	^**^	AFP	−6.392555822	^**^	INA	−2.039915212	^**^
FSTL5	−2.276301555	^*^	A2ML1	−4.325521213	^***^	TBR1	−3.109896148	^***^
ATP4A	−2.397331265	^*^	KCNH5	−2.848304727	^*^	CPS1	−2.851111003	^*^
CRABP1	−3.349924564	^**^	SPX	−2.490623817	^*^	SLC30A10	−4.590543663	^**^
KHDC1L	−3.588397669	^**^	MAGEA3	−3.016068656	^*^	BNC1	−3.674589844	^*^
RAB3B	−2.06611634	^***^	CYP2B6	2.975488322	^*^	LBP	−3.308232739	^*^
SLC13A5	−2.433197228	^***^	SOHLH1	−3.55661039	^*^	TRIM40	−2.736765196	^**^
FAM9B	−2.35516841	^*^	MEP1B	−2.120697973	^*^	PAH	−3.119569601	^***^
IGF2BP1	−2.730067985	^*^	BEST3	−2.441217072	^**^	MAGEA12	−2.240085198	^*^
INHA	−2.481472865	^***^	HOXA13	−2.248089536	^**^	ADGRF2	−2.28498311	^**^
ABCC2	−2.539707872	^**^	CNTNAP4	−3.098050369	^*^	GABRQ	−2.155966809	^*^
ETNPPL	−2.154731918	^**^	KLK2	−2.235050846	^*^	TMPRSS11A	−2.985547264	^***^
FAM163A	−2.068836158	^**^	FOXD1	−2.16123686	^**^	KCNC1	−2.191200945	^*^
HOXB9	−2.678736573	^*^	ALDOB	2.218355443	^*^	AC008763.3	2.244368079	^***^
MSMB	−2.544367684	^*^	PI3	−2.921061292	^***^	EVX1	−2.083636412	^*^
DKK1	−2.17495906	^*^	GNG4	−2.044720885	^***^	HIST1H4C	−2.297578339	^**^
HEPHL1	−2.404922517	^*^	SLC6A2	−2.027957245	^**^	ELANE	2.228845734	^***^
KRT6A	−2.767443824	^**^	KRT6C	−2.712748642	^***^	CHGB	−2.082132761	^*^
AZU1	2.289748111	^***^	HIST1H3F	−2.275440985	^*^	GRM4	−2.245814212	^**^
PATE2	−2.538391387	^***^	CHRNA9	−3.192651727	^**^	RETN	2.013746243	^***^

Abbreviation: LUAD: lung adenocarcinoma; ^*^P < 0.05; ^**^P < 0.01; ^***^P < 0.001.

### Risk model of the DEGTGs

**Figure 9:**
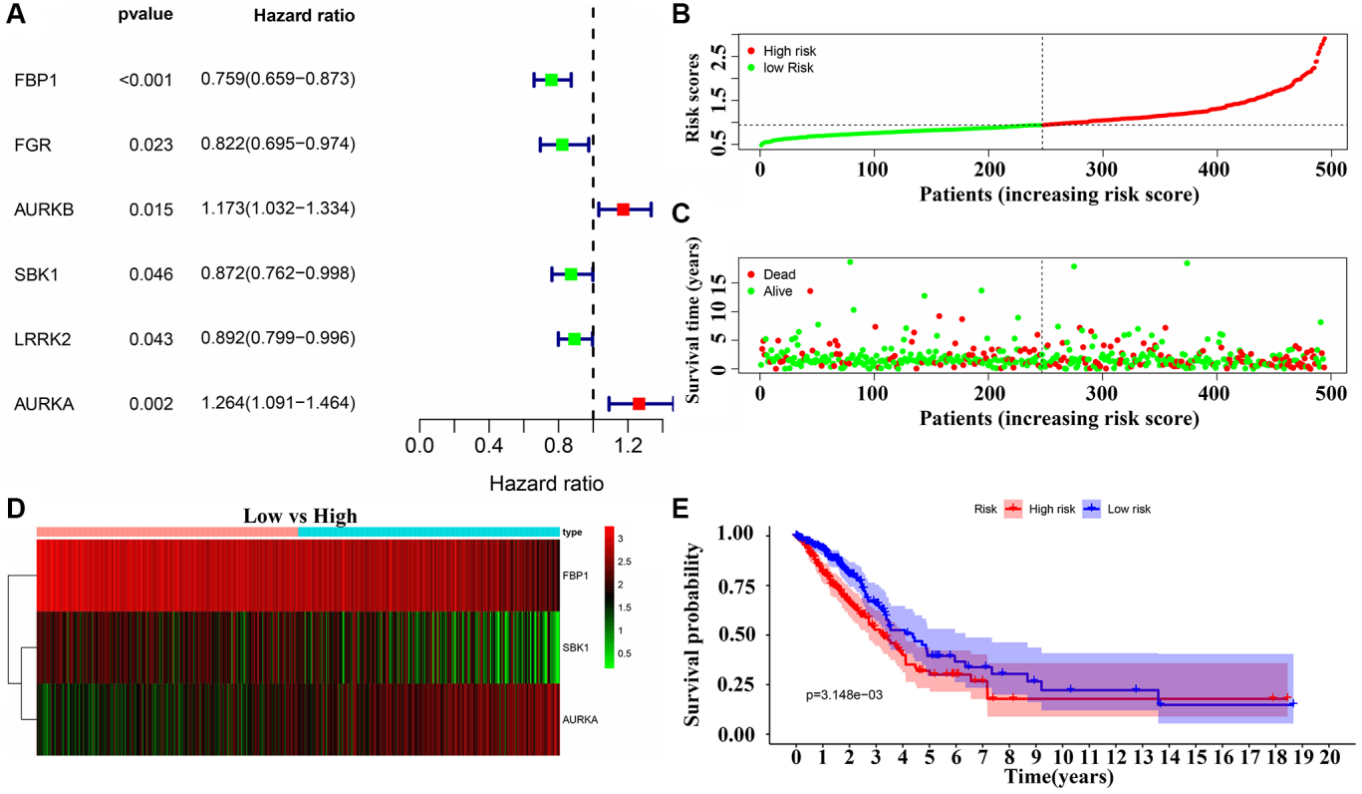
**Risk model of the DEGTGs in LUAD.** (**A**) Risk genes that affect patient prognosis in LUAD; (**B**–**E**) Patients with higher risk scores have the poor prognosis. Abbreviations: DEGTGs: differentially expressed Gefitinib target genes; LUAD: lung adenocarcinoma.

**Figure 10:**
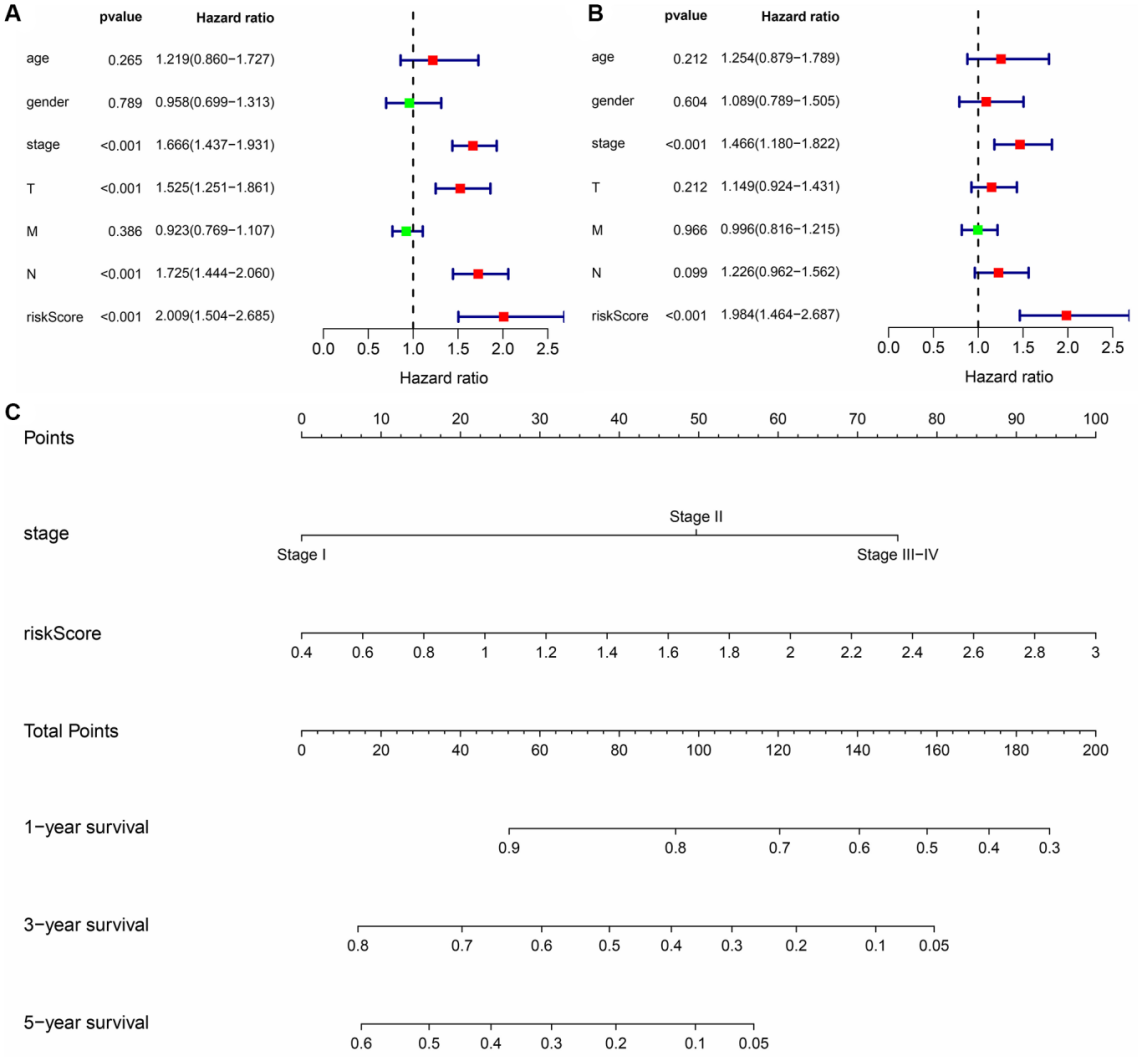
**Prognosis-related risk scores and nomogram in LUAD.** (**A**, **B**) Cox regression analysis that risk score is an independent risk factor for poor prognosis in LUAD patients; (**C**) Nomogram. Abbreviations: LUAD: lung adenocarcinoma.

Univariate Cox regression analysis revealed that the expression levels of FBP1, FGR, AURKB, SBK1, LRRK2 and AURKA were significantly correlated with the OS of LUAD (Figure 9A). Multivariate COX regression analysis and the AIC method revealed that FBP1, SBK1, and AURKA were independent predictors of OS and were the influencing factors of the risk model (Figure 9B). Moreover, we found that Gefitinib promoted FBP1 mRNA and protein expression and inhibited the SBK1 and AURKA mRNA and protein expression in PC9 cells (Supplementary Figure 1). In addition, high-risk patients had a poor prognosis with the risk model based on FBP1, SBK1, and AURKA (Figure 9C–9E). Therefore, we constructed a nomogram for Gefitinib target genes FBP1, SBK1, and AURKA (Supplementary Figure 2). Univariate and multivariate Cox regression analysis revealed that the risk score and clinical stage correlated with poor prognosis in LUAD patients (Figure 10A, 10B). Therefore, we constructed a risk model-related nomogram (Figure 10C).

### Signaling mechanisms involved in risk model

In the risk model based on FBP1, SBK1 and AURKA, the high-risk score group was enriched in the cell cycle, oocyte meiosis, RNA degradation, basal transcription factors, spliceosome, ubiquitin-mediated proteolysis, homologous recombination, mismatch repair, p53 signaling pathway, regulation of autophagy, DNA replication, base excision repair and others (Table 6).

**Table 6:** Signaling pathways in LUAD of the high-risk group in the risk model.

**Name**	**Size**	**NES**	**Nom *p***
Cell cycle	124	2.328981	0
Oocyte meiosis	112	2.1046698	0
RNA degradation	57	2.085798	0
Pyrimidine metabolism	97	2.0830867	0
Basal transcription factors	35	2.0283968	0
Spliceosome	126	2.0177684	0
Ubiquitin mediated proteolysis	133	1.9816493	0
DNA replication	36	1.9667591	0
Homologous recombination	28	1.9288028	0.00407332
Mismatch repair	23	1.9153036	0.008492569
Proteasome	44	1.912825	0.002074689
Nucleotide excision repair	44	1.8854126	0.008492569
Progesterone mediated oocyte maturation	85	1.856405	0
One carbon pool by folate	17	1.8456696	0.006060606
P53 signaling pathway	68	1.8163487	0.004219409
Purine metabolism	156	1.7766677	0.006276151
N glycan biosynthesis	46	1.7451223	0.011904762
Regulation of autophagy	35	1.6964251	0.008438818
Cysteine and methionine metabolism	34	1.6731151	0.013779528
Protein export	23	1.6567074	0.036608864
Small cell lung cancer	84	1.6488291	0.020449897
Riboflavin metabolism	15	1.6322458	0.024
Base excision repair	33	1.6144714	0.042918455

### The risk score correlated with the immune microenvironment in LUAD

**Figure 11:**
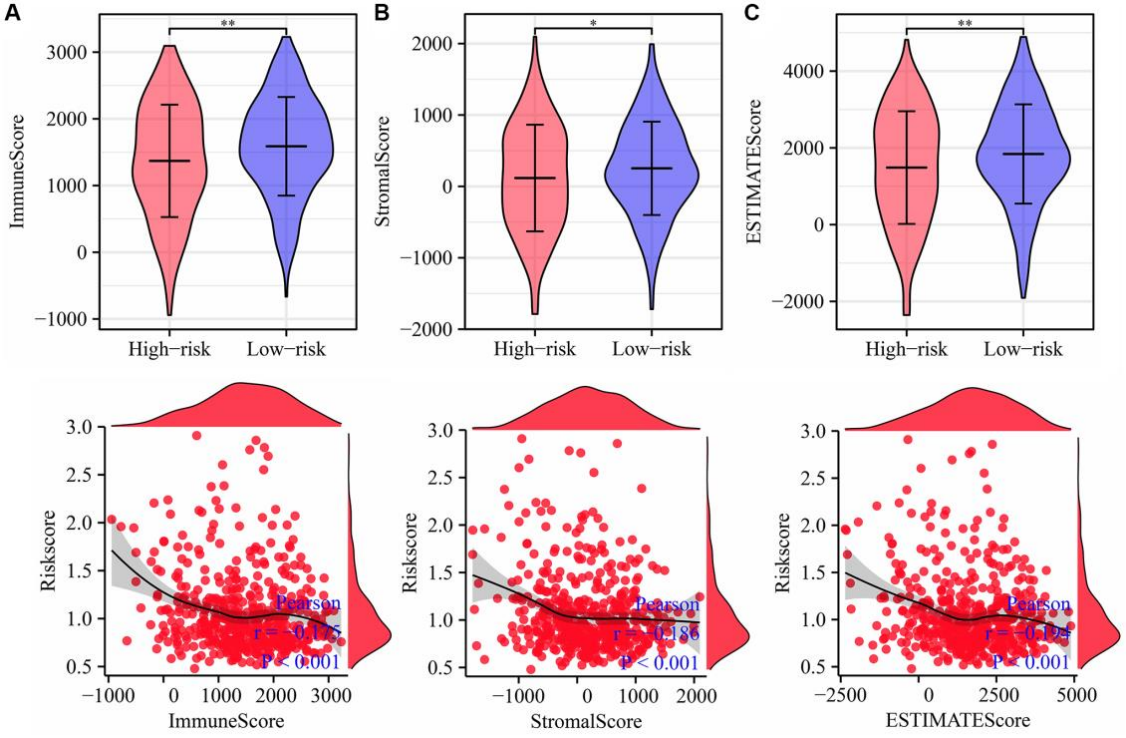
**Risk score is related to the immune, estimate, and stromal scores in LUAD.** (**A**) Immune score; (**B**) Estimate score; (**C**) Stromal score. Abbreviation: LUAD: lung adenocarcinoma.

**Figure 12:**
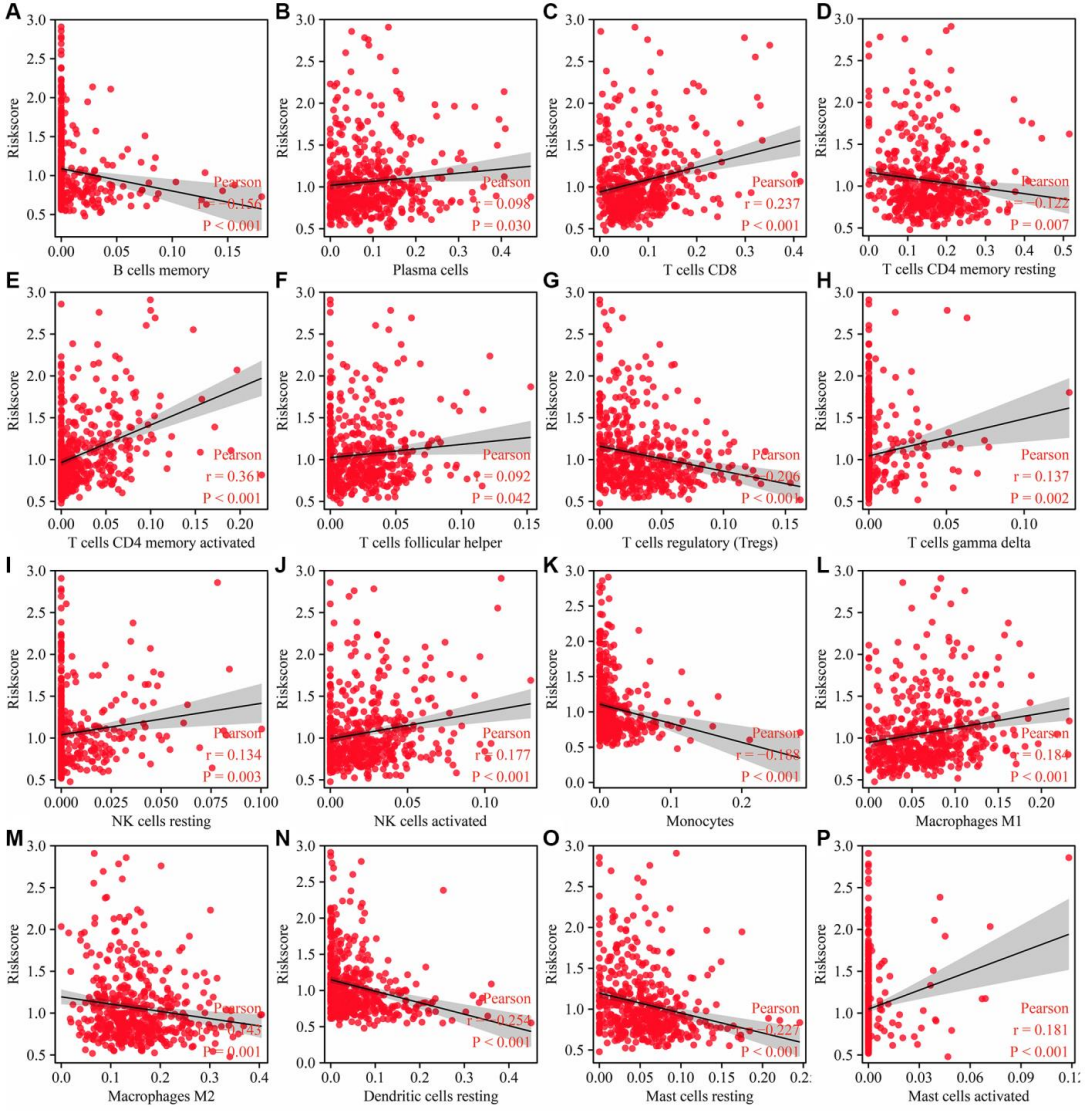
**Risk model is related to the immune cells in LUAD.** (**A**) B cells memory; (**B**) Plasma cells; (**C**) T cells CD8; (**D**) T cells CD4 memory resting; (**E**) T cells CD4 memory activated; (**F**) T cells follicular helper; (**G**) T cells regulatory (Tregs); (**H**) T cells gamma delta; (**I**) NK cells resting; (**J**) NK cells activated; (**K**) Monocytes; (**L**) Macrophages M1; (**M**) Macrophages M2; (**N**) Dendritic cells resting; (**O**) Mast cells resting; (**P**) Mast cells activated. Abbreviation: LUAD: lung adenocarcinoma.

As shown in Figure 11, the risk score was significantly correlated with the immune score, estimate score, stromal score, and immune cells, including memory B cells, plasma cells, CD8 T cells, resting CD4 memory T cells, activated CD4 memory T cells, follicular helper T cells, regulatory T cells (Tregs), gamma delta T cells, resting NK cells, activated NK cells, monocytes, M1 macrophages, M2 macrophages, resting dendritic cells, resting mast cells, and activated mast cells (Figure 12). The expression levels of naïve B cells, memory B cells, resting dendritic cells, monocytes, M1 macrophages, plasma cells, resting mast cells, gamma delta T cells, activated CD4 memory T cells, resting NK cells, resting low-risk groups were significantly different (Supplementary Figure 3).

**Figure 13:**
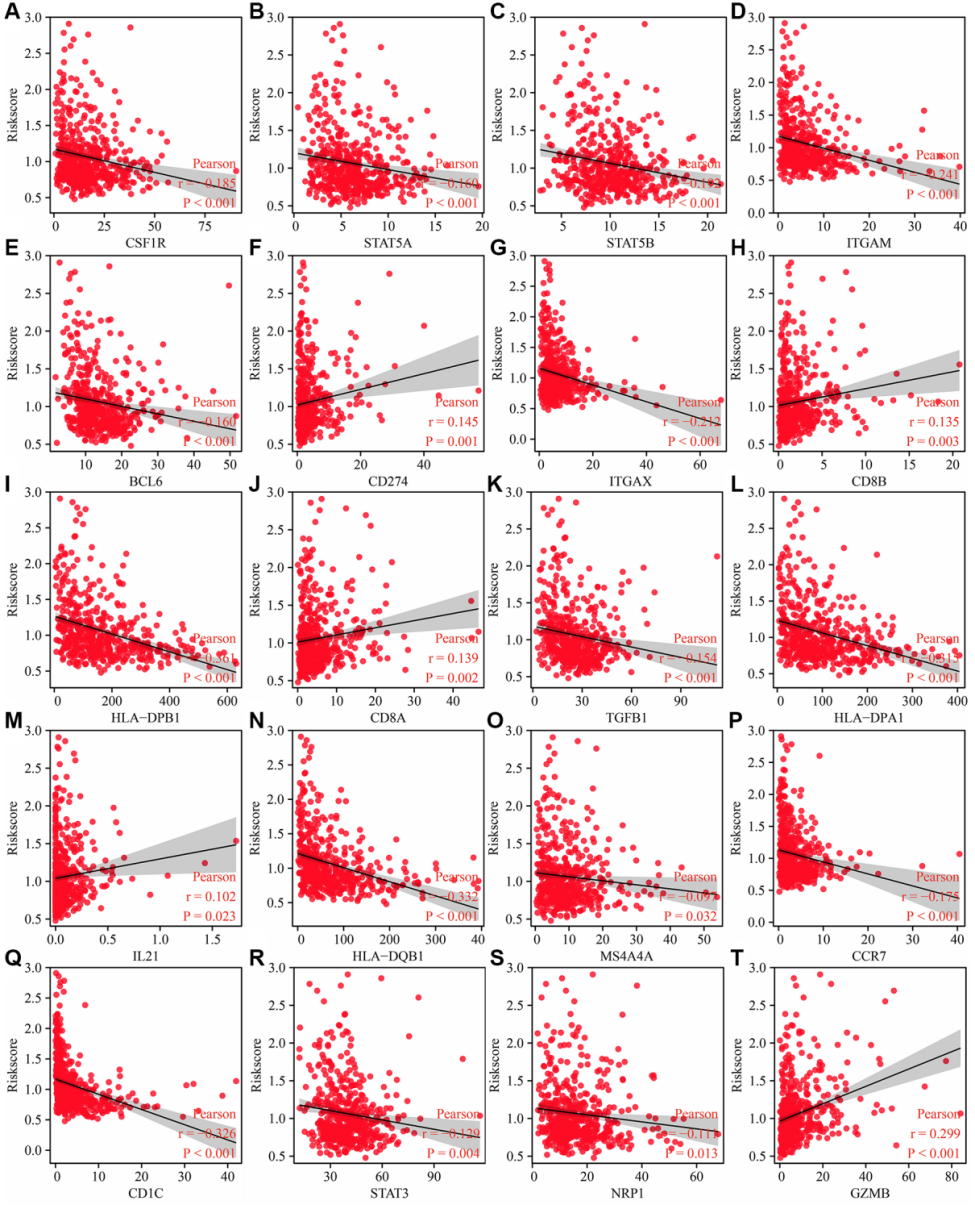
**Risk model related to the immune cell markers in LUAD.** (**A**) CSF1R; (**B**) STAT5A; (**C**) STAT5B; (**D**) ITGAM; (**E**) BCL6; (**F**) CD274; (**G**) ITGAX; (**H**) CD8B; (**I**) HLA-DPB1; (**J**) CD8A; (**K**) TGFB1; (**L**) HLA-DPA1; (**M**) IL21; (**N**) HLA-DQB1; (**O**) S4A4A; (**P**) CCR7; (**Q**) CD1C; (**R**) STAT3; (**S**) NRP1; (**T**)GZMB. Abbreviation: LUAD: lung adenocarcinoma.

The risk score was significantly correlated with immune cell markers such as CSF1R, STAT5A, STAT5B, ITGAM, BCL6, CD274 (PD-L1), ITGAX, CD8B, and others (Figure 13 and Supplementary Figure 4). In addition, immune cell markers such as CSF1R, STAT5A, STAT5B, ITGAM, BCL6, CD274, ITGAX, CD8B, CD1C, HLA-DPB1, CD8A, NRP1, GZMB, CEACAM8, HLA-DRA, KIR3DL3, IRF5, TGFB1, HLA-DPA1, IL21, HLA-DQB1, KIR2DL3, and TNF were significantly different between the high- and low-risk groups (Supplementary Figure 5).

## DISCUSSION

It is well-established that Gefitinib can improve the prognosis of patients with EGFR-mutant LUAD. Current evidence suggests that compared with chemotherapy alone, chemotherapy combined with Gefitinib could improve the PFS, ORR, and OS in LUAD patients with EGFR mutations [6]. In the present study, we found that with an increase in Gefitinib concentration, the proliferation ability of LUAD PC9 cells decreased, the apoptosis rate raised significantly, and cell migration and invasion were inhibited. Moreover, Gefitinib target genes are significantly enriched in intracellular signal transduction, positive regulation of phosphatidylinositol 3-kinase activity, positive regulation of phosphatidylinositol 3-kinase signaling, receptor signaling protein tyrosine kinase activity, kinase activity, signaling transduction, vascular endothelial growth factor-activated receptor activity, positive regulation of cell migration, vascular endothelial growth factor receptor signaling pathway, receptor complex, positive regulation of MAPK cascade, cell migration, growth factor binding, regulation of cell proliferation, negative regulation of apoptotic process, cell cycle, JNK cascade, etc. These findings indicate that Gefitinib has important anticancer effects against the progression of LUAD.

There were 100 Gefitinib target genes in the SwissTargetPrediction database, which were mainly kinases and most targets were the DEGs in LUAD tissues. The levels of Gefitinib targets AURKA, AURKB, FBP1, FGR, HCK, LRRK2, and SBK1, were associated with a poor prognosis in patients with LUAD and had diagnostic values for LUAD. Cox regression analysis revealed that the expression levels of FBP1, SBK1 and AURKA correlated with the OS and the risk model in LUAD. In PC9 cells, Gefitinib could promote FBP1 expression and inhibit SBK1 and AURKA levels. An increasing body of evidence suggests that FBP1, SBK1 and AURKA are associated with lung cancer progression [10–15]. For instance, FBP1 was found to be downregulated in lung cancer tissues and cells. Decreased levels of FBP1 expression were associated with poor prognosis in lung cancer patients. Activation of FBP1 expression could inhibit glucose uptake and lactate production, induce oxygen consumption capacity, and inhibit lung cancer cell proliferation and invasion under hypoxic conditions *in vitro* and lung cancer growth *in vivo* [10]. High expression of AURKA correlated with shorter OS in lung cancer patients. Inhibition of AURKA expression reduced lung cancer cell growth and enhanced the sensitivity of lung cancer cells to radiation [13]. Upon inhibition of long non-coding RNA ENST000001520 in BEAS-2B cells, a notable decrease is observed in cell migration and proliferation abilities, accompanied by an increase in apoptosis capabilities. These changes can be attributed to the downregulation of SBK1 and SOCS3 mRNA and protein expression [14]. This finding indicated that Gefitinib could affect FBP1, SBK1 and AURKA expression to inhibit LUAD progression.

In recent years, risk models have been used to assess the prognosis of patients with LUAD [2, 16–18]. For example, XRCC4, XRCC5 and XRCC6 were identified as the risk factors affecting the prognosis of patients with LUAD. A risk model based on XRCC4, XRCC5 and XRCC6 showed that the risk score was related to the prognosis, gender, clinical stage, T stage, lymph node metastasis and metastasis of LUAD patients and was an independent risk factor for the prognosis of LUAD patients [2]. The risk model and nomogram constructed based on Gefitinib target genes FBP1, SBK1, and AURKA were risk factors associated with poor prognosis of LUAD patients. The risk score was significantly correlated with the immune score, estimate score, stromal score, memory B cells, plasma cells, CD8T cells, resting CD4 memory T cells, activated CD4 memory T cells, follicular helper T cells, Tregs, gamma delta T cells, resting NK cells, activated NK cells, monocytes, M1 macrophages, M2 macrophages, resting dendritic cells, resting mast cells, and activated mast cells, and with CSF1R, STAT5A, STAT5B, ITGAM, BCL6, PD-L1, ITGAX, CD8B, and other immune cell marker levels.

Overall, we explored the roles of Gefitinib against LUAD progression using bioinformatics and cellular experiments and found that the risk model constructed based on the Gefitinib target genes FBP1, SBK1, and AURKA was associated with a dismal prognosis and was closely related to the immune microenvironment. However, the relationship between EGFR mutations and Gefitinib target genes (FBP1, SBK1, and AURKA) warrants further investigation, and cancer tissues were collected to confirm the relationship between the risk model and nomogram and the prognosis of LUAD patients in the future. In addition, we will further investigate the impact of downregulating or promoting FBP1, SBK1, and AURKA expression in PC9 cells on lung adenocarcinoma in the future. In general, Gefitinib could inhibit the proliferation, migration and invasion, promote the apoptosis of LUAD PC9 cells and the expression of FBP1, and inhibit the expression of SBK1 and AURKA. High-risk LUAD patients based on the FBP1, SBK1, and AURKA had a poor prognosis. Our risk model and nomogram based on the FBP1, SBK1 and AURKA were associated with poor prognosis and immune cell infiltration levels in LUAD patients, suggesting their significant value in evaluating the prognosis of LUAD patients.

## CONCLUSIONS

The present results indicate that Gefitinib could inhibit the proliferation, migration and invasion, promote the apoptosis of LUAD PC9 cells and the expression of FBP1, and inhibit the expression of SBK1 and AURKA. High-risk LUAD patients based on the FBP1, SBK1, and AURKA had a poor prognosis. Our risk model and nomogram based on the FBP1, SBK1 and AURKA were associated with poor prognosis and immune cell infiltration levels in LUAD patients.

## MATERIALS AND METHODS

### PC9 cell culture and construction of cell models

The PC9 cell line was cultured in the RPMI-1640 with 10% fetal bovine serum at 37°C in an incubator with 5% CO_2_. The PC9 cells were cultured in 6-well plates for 24 h in an incubator, then 0, 0.002, 0.02, 0.2, and 2 μM of Gefitinib were added.

### Cell growth

The effects of Gefitinib on the proliferation and apoptosis of PC9 cells were detected by CCK-8 and flow cytometry. The experimental steps of CCK-8 were as follows: cell counting and 96-well plate plating, and 0, 0.002, 0.02, 0.2 and 2 μM Gefitinib were added to incubate cells. The CCK-8 solution at 24, 48, and 72 h incubation, and reading after 1 h incubation to appraisal the absorbance values of each group. The flow cytometry experiment steps were as follows: cell counting, and 6-well plate plating. 0, 0.002, 0.02, 0.2, and 2 μM Gefitinib were added to incubate cells, and stained according to the manufacturer’s instructions. The apoptotic PC9 cells were immediately detected using the FACS Caliber II Sorter and the Cell Quest FACS system (BD Biosciences, USA). The data were analyzed using FlowJo software (Version: 7.6.5).

### Cell migration and invasion

In 6 well plate, the clean tip of pipette is used to create “wound” when cells form confluent monolayer, and cells cultured in RPMI-1640 were supplemented with 0% fetal bovine serum. Transfected cells were used to capture the image of wound edge with optical microscope at 0 h. After 24 h of incubation, the cell images of the same area were captured for measurement, and the wound healing rate was calculated [19]. Transfected cells were investigated for transwell migration. The basal lateral chamber was balanced with 600 μL RPMI-1640 medium containing 10% fetal bovine serum, while the apical chamber was added with 200 μL PC9 cell suspension without FBS. After 24 h of incubation at 37°C and 5% CO_2_. According to the manufacturer's agreement of transwell System, fix the transwell chamber with 5% paraformaldehyde for 15 min, and then dye it with 0.1% crystal violet for 10 min. Transmembrane cells were calculated under inverted fluorescence microscope [19].

### Acquisition and identification of Gefitinib target genes

The secondary structure of Gefitinib was obtained from the official website of the PubChem database, and then the target genes of Gefitinib were obtained from the SwissTargetPrediction database using the structure of Gefitinib. The transcriptome data of 59 normal tissues and 535 LUAD tissues were downloaded from the official website of The Cancer Genome Atlas (TCGA) database, and the gene expression data of Gefitinib targets in normal tissues and cancer tissues were extracted and analyzed using the Limma package.

### Biological functions, signaling mechanisms and PPI network of DEGTGs

A *P*-value < 0.05 was used to screen for significant DEGTGs in LUAD tissues. The DEGTGs underwent GO and KEGG analyses to investigate the biological functions and signaling mechanisms of Gefitinib target genes. A PPI network of the DEGTGs was constructed in the STRING database. The PPI network was visualized by Cytoscape software, and the key genes were selected by the degree method using the CytoHubba plugin [19, 20].

### Construction of a nomogram of prognostic-related Gefitinib target genes

The data of DEGTGs were merged with the prognostic information of LUAD patients, and patients with incomplete prognostic information were excluded. The patients were stratified based on the median value of the DEGTGs, and the relationship between the expression level of DEGTGs and prognosis was identified using survival analysis. The diagnostic value of prognostic genes AURKA, AURKB, FBP1, FGR, HCK, LRRK2, and SBK1 for LUAD was assessed using the ROC analysis. A prognostic nomogram of the Gefitinib target genes was constructed based on survival and ROC analysis results.

### The roles of Gefitinib target gene subgroups in LUAD using consensus clustering

The gene expression data of AURKA, AURKB, FBP1, FGR, HCK, LRRK2, and SBK1 in LUAD tissues from TCGA were extracted. The Consensus ClusterPlus package clustered LUAD patients and determined the optimal *k* value to group the LUAD patients. Principal component analysis (PCA) was used to explore the differences between subgroups constructed based on the AURKA, AURKB, FBP1, FGR, HCK, LRRK2, and SBK1. The differentially expressed genes (DEGs) in the tissues of the two groups of LUAD patients were identified using the screening criteria logFC (>2 or <−2) and FDR < 0.05. The biological functions and signaling mechanisms involved in DEGs in the subgroups of Gefitinib target genes were explored using GO and KEGG analyses. The relationship between DEGs in the subgroups of Gefitinib target genes and OS of LUAD patients was analyzed using COX regression analysis, and the expression of prognostic genes in normal lung tissues and LUAD tissues was visualized.

### Construction of risk model based on the DEGTGs

Univariate Cox regression analysis was used to reveal the relationship between the gene expression levels of DEGTGs AURKA, AURKB, FBP1, FGR, HCK, LRRK2, and SBK1 and OS in patients with LUAD, with *P* < 0.05 as the screening criteria. Then, the relationship between the expression levels of FBP1, FGR, AURKB, SBK1, LRRK2, and AURKA and the OS of LUAD patients was analyzed using multivariate COX regression analysis and the Akaike information criterion (AIC) method. The effects of Gefitinib on AURKA, FBP1, and SBK1 were assessed by qRT-PCR and western blotting, and a risk model was established based on these results.

### qRT-PCR

Trizol (Thermo Fisher Scientific, China) was used to isolate total RNA from the PC9 cells after supplementing Gefitinib, and total RNAs were reverse-transcribed into cDNA using the Revertra Ace qPCR RT Kit (Toyobo life science, Japan). The UltraSYBR mixture (cwbio, China) was used to complete the PCR cycles using a StepOne Plus Real-Time PCR System (ABI, USA). The change in relative AURKA, FBP1 and SBK1 mRNA expression levels were calculated using the 2^ΔΔct^ method. The primer sequences of AURKA, FBP1 and SBK1, and β-actin used were as follows. AURKA forward: 5′-GTGGAGCATCAGCTCAGAAGA-3′ and reverse 5′-ACTGTTCCAAGTGGTGCATA-3′; FBP1 forward: 5′-ATCGATTGCCTTGTGTCCGT-3′ and reverse 5′-AAGCAGTTGACCCCACAGTC-3′; SBK1 forward: 5′-ACGTCACCAAGCA CTACGAA-3′ and reverse 5′-GTGATGCTCACCTCCCGTAG-3′; β-actin forward 5′-ACTCTTCCAGCCTTCCTTCC-3′ and reverse 5′-CGTCA TACTCCTGCTTGCTG-3′.

### Western blotting

Total protein was isolated from PC9 cells by using cell lysate after Gefitinib supplementation. Protein gel electrophoresis was performed after quantitative analysis using the BCA method. After electroporation, the PVDF film was cut and incubated overnight at low temperature with the primary antibody. The second antibody was incubated, and the protein was exposed the following day. The primary antibodies used, along with their respective concentrations, were the 1:3000 FBP1 (Proteintech, China), 1:1000 SBK1 (HUABIO, China), 1:1000 AURKA (HUABIO, China), and 1:10000 GAPDH (Proteintech, China).

### Construction of a nomogram of risk model

Univariate and multivariate COX regression analysis was used to reveal the relationship between the age, gender, clinical stage, T stage, N stage, M stage, risk score, and prognosis of cancer patients. Finally, a risk model related to the nomogram was constructed.

### Signaling mechanisms of the risk model

Gene Set enrichment analysis (GSEA) is a commonly used method to reveal the functions and mechanisms involved in the risk models [21–23]. In our study, we divided the gene expression data of LUAD patients into high- and low-risk groups. The impact of high- and low-risk groups was analyzed on each gene set to reveal the signaling mechanism involved in the risk model in the GSEA software [21–23].

### Analysis of the relationship between the risk model and the LUAD immune microenvironment

The gene expression data in 535 LUAD tissues were immunoscored by using the CIBERSORT and estimate methods. Correlation analysis was used to reveal the relationship between the risk score and the levels of immune cells, stromal score, immune score, estimate score and immune cell markers in LUAD. The levels of immune cells, stromal score, immune score, estimate score, and immune cell markers in the high- and low-risk groups were explored in LUAD.

### Statistical analysis

The significance of the effect of Gefitinib on LUAD cell growth and migration and the difference in the expression of the immune cells, stromal score, immune score, estimate score and immune cell markers in high- and low-risk groups were identified using the Student’s *t*-test. Survival and Cox regression analyses were used to assess the prognostic value of Gefitinib target genes. The diagnostic values of Gefitinib target genes for LUAD were identified using the ROC analysis. The AUC close to 1 was associated with a good diagnostic value. A *P*-value < 0.05 was statistically significant.

### Availability of data and materials

The data were acquired from publicly available databases, and additional data could be obtained from the corresponding author upon reasonable request.

## Supplementary Materials

Supplementary Figures

Supplementary Tables
