# Supplementary material for: A novel risk model of three gefitinib-related genes FBP1, SBK1 and AURKA is related to the immune microenvironment and is predicting prognosis of lung adenocarcinoma patients
Source: Aging (Albany NY). 2023 Sep 21;15(18):9633–60. doi: 10.18632/aging.205040 (PMC10564433; doi:10.18632/aging.205040)
Supplement: Supplementary Figures [file aging-15-205040-s001.pdf]

SUPPLEMENTARY FIGURES

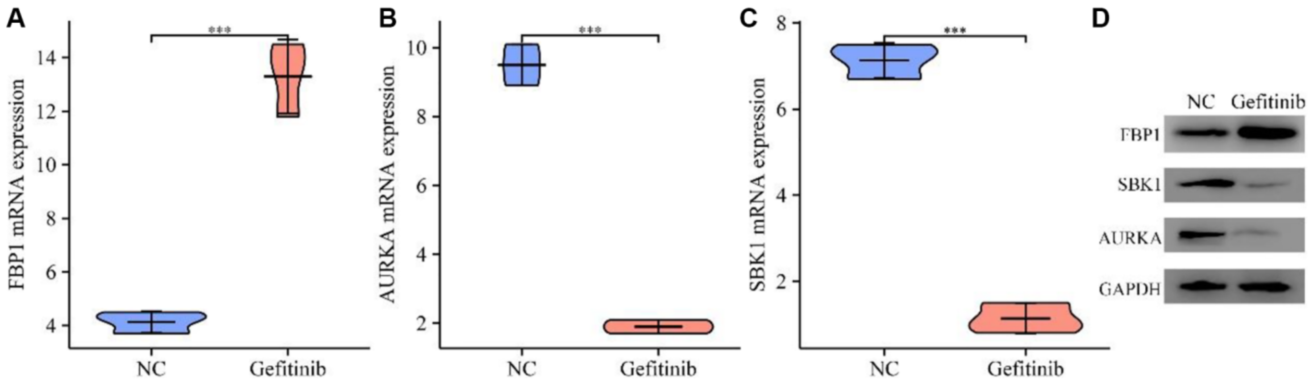

Supplementary Figure 1. Gefitinib could regulate the levels of FBP1, AURKA, and SBK1 in PC9 cells. (**A**–**C**) mRNA levels; (**D**) Protein levels.

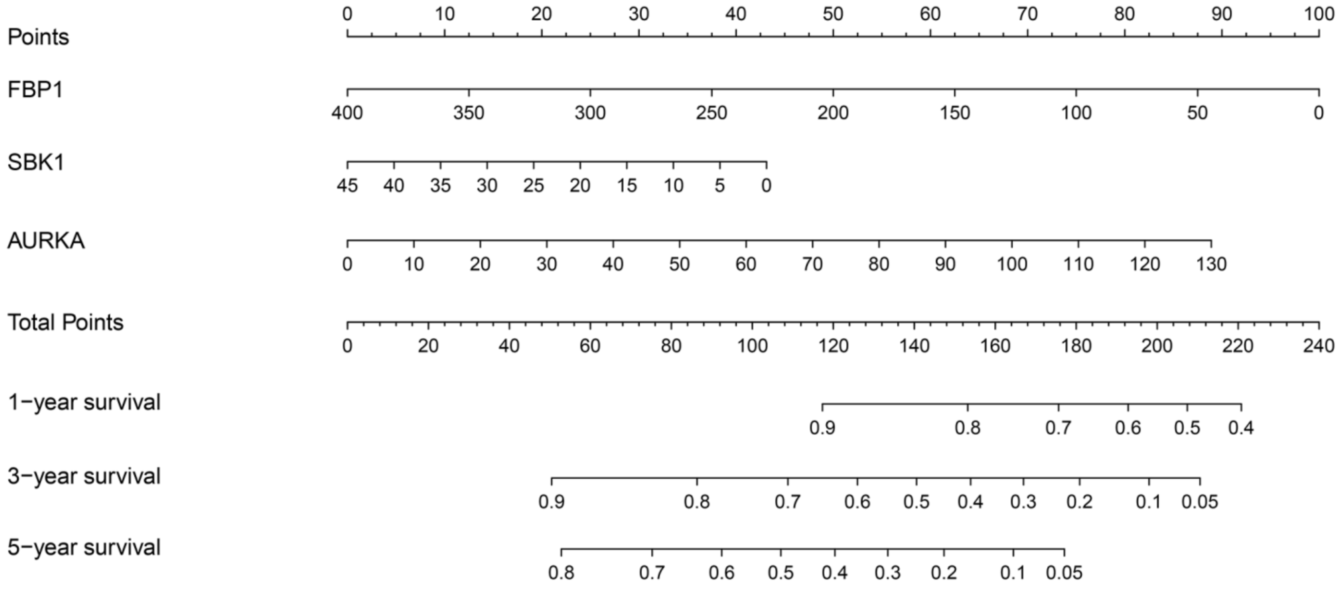

Supplementary Figure 2. Nomogram of risk model genes.

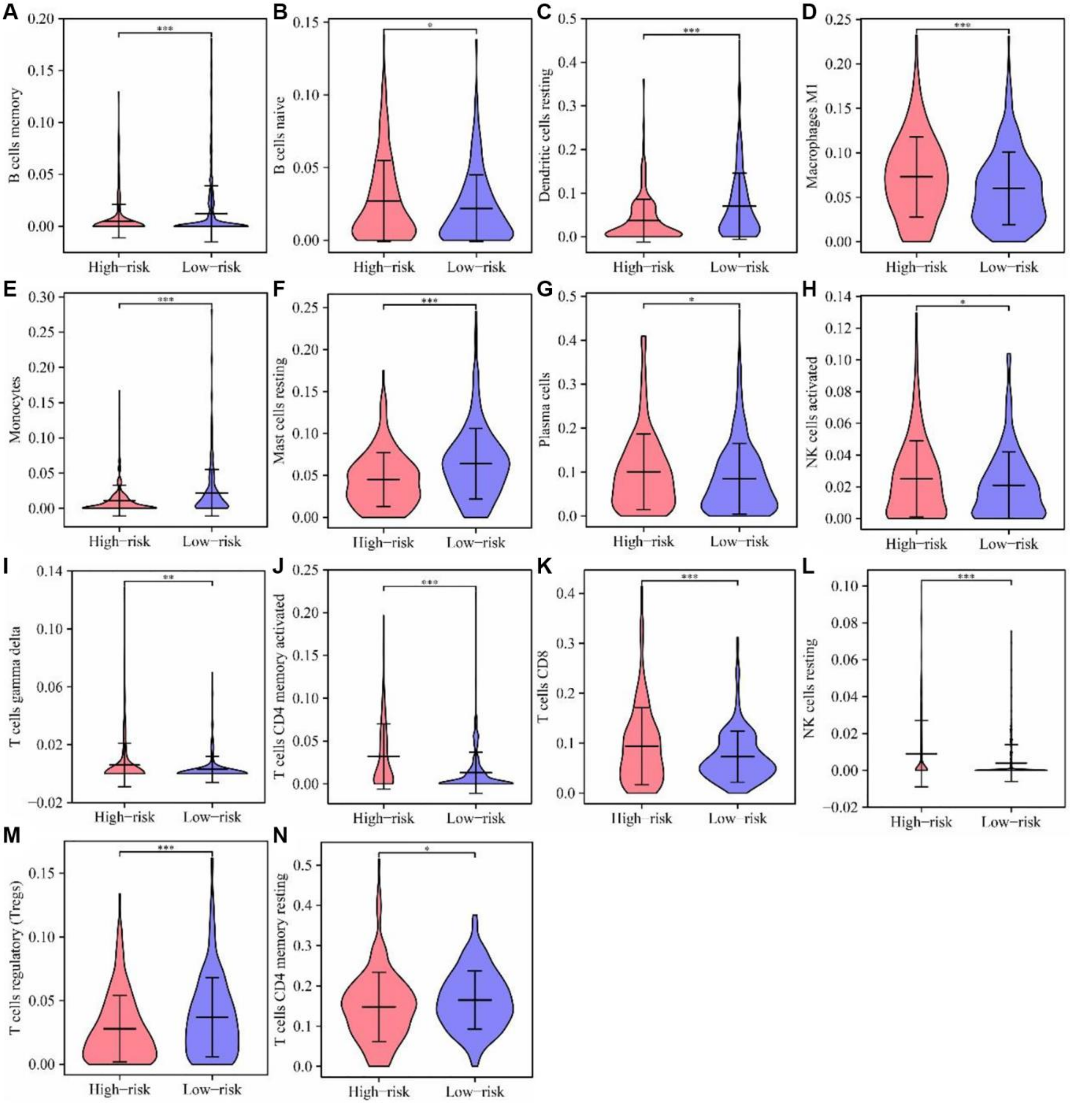

Supplementary Figure 3. The levels of immune cells in high-risk and low-risk in the risk model. (**A**) B cells memory; (**B**) B cells naive; (**C**) Dendritic cells resting; (**D**) Macrophages M1; (**E**) Monocytes; (**F**) Mast cells resting; (**G**) Plasma cells; (**H**) NK cells activated; (**I**) T cells gamma delta; (**J**) T cells CD4 memory activated; (**K**) T cells CD8; (**L**) NK cells resting; (**M**) Regulatory T cells (Tregs); (**N**) T cells CD4 memory resting.

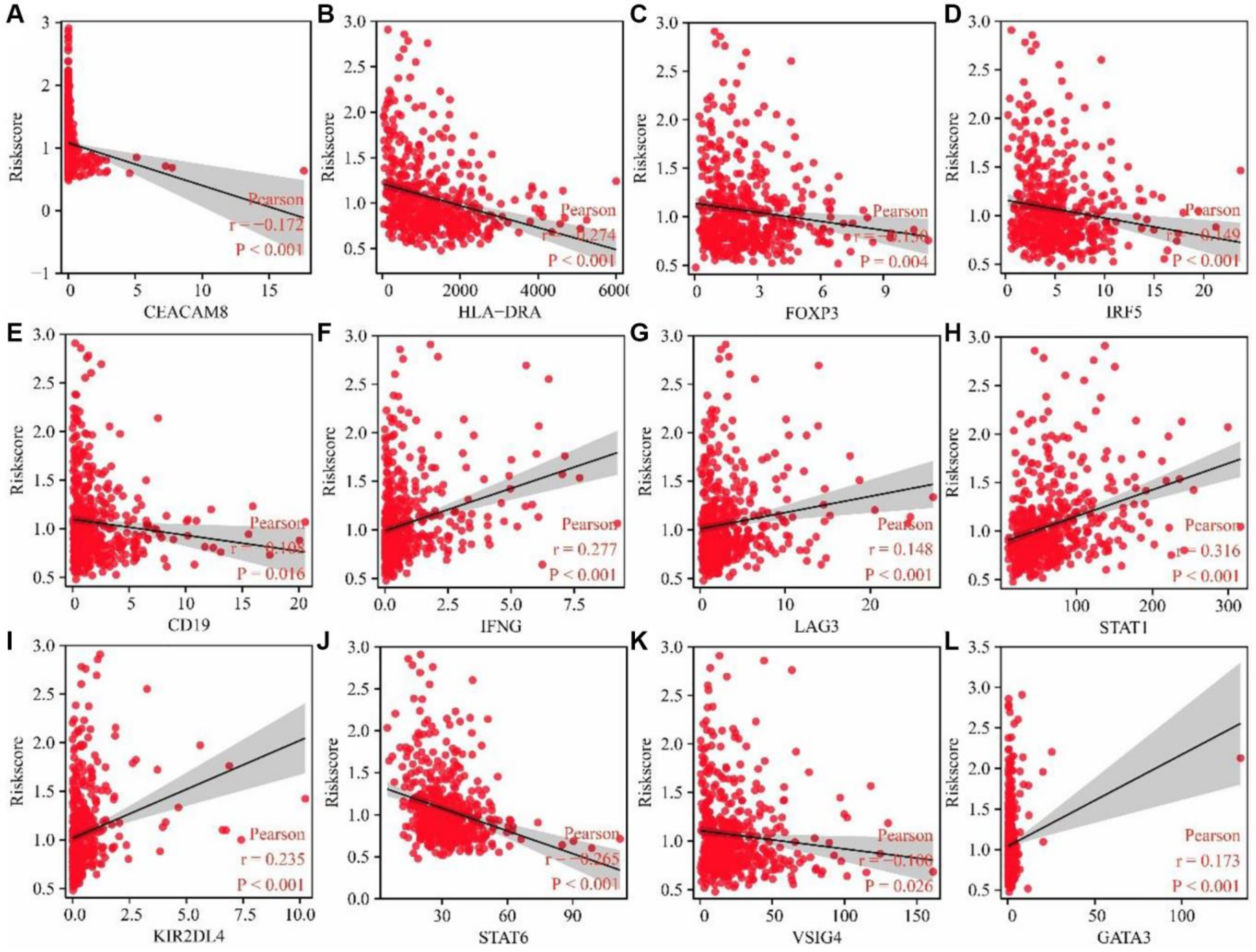

Supplementary Figure 4. Risk model related to the immune cell markers in LUAD. (**A**) CEACAM8; (**B**) HLA-DPA1; (**C**) FOXP3; (**D**) IRF5; (**E**) CD19; (**F**) IFNG; (**G**) LAG3; (**H**) STAT1; (**I**) KIR2DL4; (**J**) STAT6; (**K**) VSIG4; (**L**) GATA3. Abbreviation: LUAD: lung adenocarcinoma.

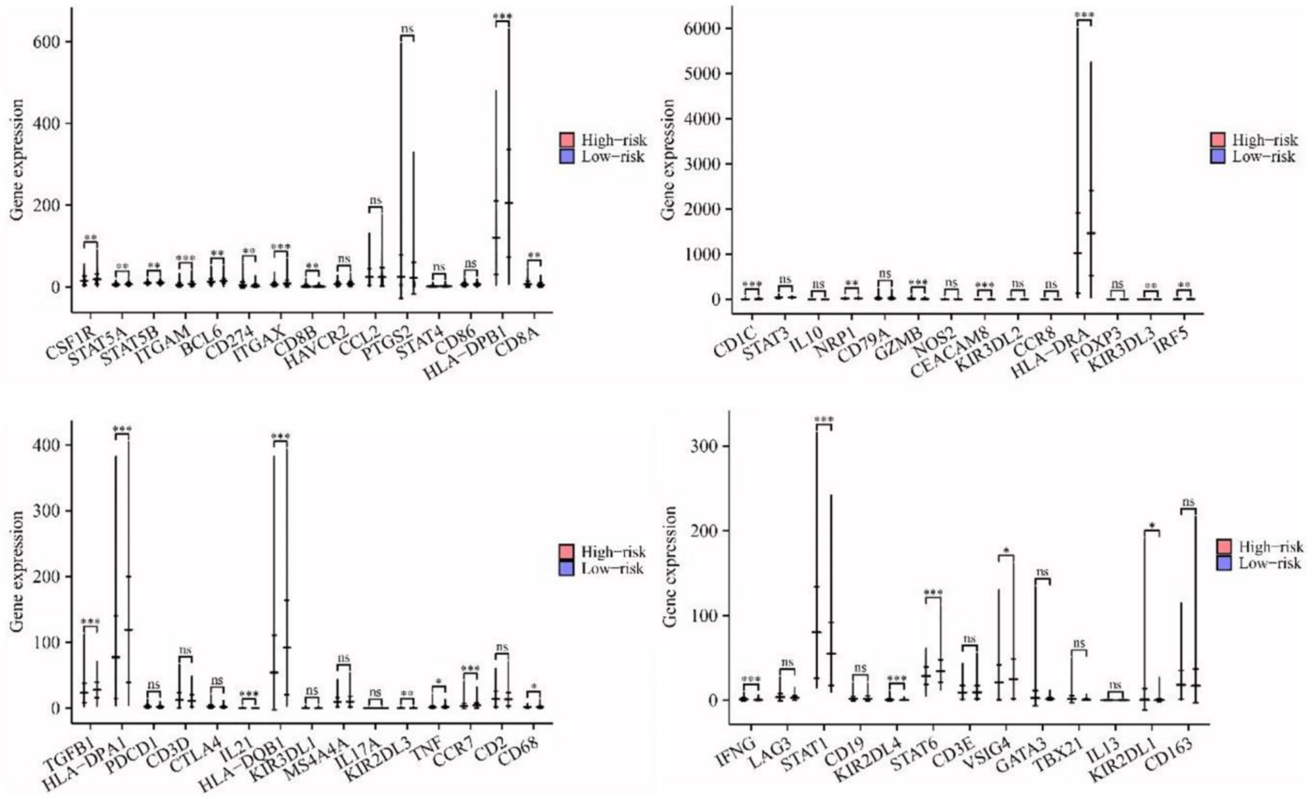

Supplementary Figure 5. Cell markers in high- and low-risk groups.
