# Supplementary material for: A novel risk model of three gefitinib-related genes FBP1, SBK1 and AURKA is related to the immune microenvironment and is predicting prognosis of lung adenocarcinoma patients
Source: Aging (Albany NY). 2023 Sep 21;15(18):9633–60. doi: 10.18632/aging.205040 (PMC10564433; doi:10.18632/aging.205040)
Supplement: Supplementary Tables [file aging-15-205040-s002.pdf]

SUPPLEMENTARY TABLES

Supplementary Table 1. Functions of the DEGTGs.

GO	Term	Category	Count	*P*
MF	GO:0005524	ATP binding	83	4.55E-86
BP	GO:0046777	Protein autophosphorylation	47	1.67E-71
MF	GO:0004672	Protein kinase activity	46	1.02E-53
BP	GO:0006468	Protein phosphorylation	47	1.92E-50
MF	GO:0004674	Protein serine/threonine kinase activity	42	7.15E-46
BP	GO:0018108	Peptidyl-tyrosine phosphorylation	32	8.93E-43
MF	GO:0004713	Protein tyrosine kinase activity	26	1.76E-33
BP	GO:0007169	Transmembrane receptor protein tyrosine kinase signaling pathway	22	1.41E-29
MF	GO:0004714	Transmembrane receptor protein tyrosine kinase activity	16	8.76E-26
BP	GO:0038083	Peptidyl-tyrosine autophosphorylation	16	2.44E-25
MF	GO:0004715	Non-membrane spanning protein tyrosine kinase activity	14	3.25E-20
BP	GO:0043552	Positive regulation of phosphatidylinositol 3-kinase activity	11	2.01E-16
BP	GO:0035556	Intracellular signal transduction	22	3.37E-16
CC	GO:0031234	Extrinsic component of cytoplasmic side of plasma membrane	12	1.10E-14
BP	GO:0014068	Positive regulation of phosphatidylinositol 3-kinase signaling	12	1.57E-14
MF	GO:0004716	Receptor signaling protein tyrosine kinase activity	7	2.43E-12
BP	GO:0000165	MAPK cascade	16	2.50E-12
BP	GO:0043406	Positive regulation of map kinase activity	10	1.16E-11
MF	GO:0005515	Protein binding	73	2.03E-11
BP	GO:0018105	Peptidyl-serine phosphorylation	12	2.55E-11
MF	GO:0005088	Ras guanyl-nucleotide exchange factor activity	11	2.32E-10
BP	GO:0010863	Positive regulation of phospholipase C activity	6	3.24E-10
BP	GO:0001934	Positive regulation of protein phosphorylation	11	6.53E-10
CC	GO:0005829	Cytosol	40	1.71E-09
MF	GO:0016301	Kinase activity	13	2.16E-09
BP	GO:0014066	Regulation of phosphatidylinositol 3-kinase signaling	9	4.47E-09
BP	GO:0007165	Signal transduction	24	5.50E-09
BP	GO:0048013	Ephrin receptor signaling pathway	9	9.80E-09
MF	GO:0005021	Vascular endothelial growth factor-activated receptor activity	5	1.88E-08
BP	GO:0046854	Phosphatidylinositol phosphorylation	9	1.99E-08
MF	GO:0046934	Phosphatidylinositol-4,5-bisphosphate 3-kinase activity	8	2.12E-08
BP	GO:0045087	Innate immune response	15	2.23E-08
BP	GO:0030335	Positive regulation of cell migration	11	2.38E-08
CC	GO:0005737	Cytoplasm	49	2.58E-08
BP	GO:0000187	Activation of mapk activity	9	5.54E-08
BP	GO:0048010	Vascular endothelial growth factor receptor signaling pathway	8	6.33E-08
CC	GO:0043235	Receptor complex	9	1.13E-07
BP	GO:0043410	Positive regulation of mapk cascade	8	1.44E-07
BP	GO:0016477	Cell migration	10	1.73E-07
MF	GO:0019838	Growth factor binding	6	1.89E-07
BP	GO:2000145	Regulation of cell motility	6	2.35E-07
BP	GO:0042127	Regulation of cell proliferation	10	3.21E-07
BP	GO:0043066	Negative regulation of apoptotic process	14	3.37E-07
MF	GO:0004707	MAP kinase activity	5	5.24E-07

MF	GO:0000287	Magnesium ion binding	10	6.99E-07
BP	GO:0048015	Phosphatidylinositol-mediated signaling	8	9.16E-07
MF	GO:0005102	Receptor binding	12	1.20E-06
MF	GO:0042803	Protein homodimerization activity	16	2.35E-06
BP	GO:0070374	Positive regulation of ERK1 and ERK2 cascade	9	2.40E-06
BP	GO:0023014	Signal transduction by protein phosphorylation	6	2.44E-06
CC	GO:0005634	Nucleus	46	2.64E-06
BP	GO:0008284	Positive regulation of cell proliferation	13	2.94E-06
BP	GO:0035924	Cellular response to vascular endothelial growth factor stimulus	5	4.57E-06
BP	GO:0008360	Regulation of cell shape	8	5.90E-06
MF	GO:0004702	Receptor signaling protein serine/threonine kinase activity	6	6.07E-06
CC	GO:0005886	Plasma membrane	38	7.39E-06
BP	GO:0007049	Cell cycle	9	1.17E-05
MF	GO:0019903	Protein phosphatase binding	6	1.43E-05
BP	GO:0001525	Angiogenesis	9	1.43E-05
CC	GO:0005887	Integral component of plasma membrane	20	1.52E-05
MF	GO:0042802	Identical protein binding	15	1.56E-05
BP	GO:0031663	Lipopolysaccharide-mediated signaling pathway	5	1.80E-05
MF	GO:0005003	Ephrin receptor activity	4	1.84E-05
BP	GO:0034446	Substrate adhesion-dependent cell spreading	5	3.60E-05
CC	GO:0045121	Membrane raft	8	4.27E-05
BP	GO:0010976	Positive regulation of neuron projection development	6	7.83E-05
BP	GO:2000249	Regulation of actin cytoskeleton reorganization	4	9.01E-05
BP	GO:0007254	JNK cascade	5	9.93E-05
BP	GO:0043547	Positive regulation of GTPase activity	12	1.02E-04

Abbreviations: DEGTGs: differentially expressed gefitinib target genes; GO: gene ontology; BP: biological process; MF: molecular function; CC: cell component.

Supplementary Table 2. Functions involved in subgroups of the DEGTGs in LUAD.

GO	ID	Description	*P*	Count
BP	GO:0051883	Killing of cells in other organism involved in symbiotic interaction	5.01131E-07	4
BP	GO:0051818	Disruption of cells of other organism involved in symbiotic interaction	8.67793E-07	4
BP	GO:0019730	Antimicrobial humoral response	1.27764E-06	7
BP	GO:0070942	Neutrophil mediated cytotoxicity	1.03059E-05	3
BP	GO:0001963	Synaptic transmission, dopaminergic	1.09352E-05	4
BP	GO:0051873	Killing by host of symbiont cells	1.87715E-05	3
BP	GO:0003002	Regionalization	2.82067E-05	9
BP	GO:0051852	Disruption by host of symbiont cells	3.08568E-05	3
BP	GO:0032225	Regulation of synaptic transmission, dopaminergic	4.71641E-05	3
BP	GO:0042035	Regulation of cytokine biosynthetic process	0.000163404	5
BP	GO:0031640	Killing of cells of other organism	0.000174369	4
BP	GO:0044364	Disruption of cells of other organism	0.000174369	4
BP	GO:0021953	Central nervous system neuron differentiation	0.000176276	6
BP	GO:0007389	Pattern specification process	0.000176336	9
BP	GO:0001505	Regulation of neurotransmitter levels	0.000192111	8
BP	GO:0006836	Neurotransmitter transport	0.000209819	7
BP	GO:0042089	Cytokine biosynthetic process	0.000233028	5

BP	GO:0042107	Cytokine metabolic process	0.000241968	5
BP	GO:2001025	Positive regulation of response to drug	0.00026536	3
BP	GO:0061844	Antimicrobial humoral immune response mediated by antimicrobial peptide	0.000327683	4
BP	GO:0009954	Proximal/distal pattern formation	0.000360573	3
BP	GO:0015893	Drug transport	0.000408537	6
BP	GO:0050829	Defense response to Gram-negative bacterium	0.000667059	4
BP	GO:0050804	Modulation of chemical synaptic transmission	0.000767437	8
BP	GO:0019835	Cytolysis	0.000769755	3
BP	GO:0099177	Regulation of trans-synaptic signaling	0.000778925	8
BP	GO:0021877	Forebrain neuron fate commitment	0.00087928	2
BP	GO:0051583	Dopamine uptake involved in synaptic transmission	0.001071537	2
BP	GO:0051934	Catecholamine uptake involved in synaptic transmission	0.001071537	2
BP	GO:0006959	Humoral immune response	0.001112298	7
BP	GO:0023061	Signal release	0.001114365	8
BP	GO:0007631	Feeding behavior	0.001117428	4
BP	GO:0060986	Endocrine hormone secretion	0.001235019	3
BP	GO:0021903	Rostrocaudal neural tube patterning	0.00128209	2
BP	GO:0015696	Ammonium transport	0.001432217	4
BP	GO:0021794	Thalamus development	0.001510774	2
BP	GO:2000052	Positive regulation of non-canonical Wnt signaling pathway	0.001510774	2
BP	GO:0051817	Modification of morphology or physiology of other organism involved in symbiotic interaction	0.001637445	4
BP	GO:0070268	Cornification	0.001637445	4
BP	GO:0030900	Forebrain development	0.001641544	7
BP	GO:0015872	Dopamine transport	0.001656262	3
BP	GO:0021879	Forebrain neuron differentiation	0.001749975	3
BP	GO:0002523	Leukocyte migration involved in inflammatory response	0.001757427	2
BP	GO:0032274	Gonadotropin secretion	0.002021886	2
BP	GO:0045073	Regulation of chemokine biosynthetic process	0.002021886	2
BP	GO:0090494	Dopamine uptake	0.002021886	2
BP	GO:2000095	Regulation of Wnt signaling pathway, planar cell polarity pathway	0.002021886	2
BP	GO:0042033	Chemokine biosynthetic process	0.002303989	2
BP	GO:0050755	Chemokine metabolic process	0.002303989	2
BP	GO:0090493	Catecholamine uptake	0.002303989	2
BP	GO:0042268	Regulation of cytolysis	0.002603576	2
BP	GO:0060850	Regulation of transcription involved in cell fate commitment	0.002603576	2
BP	GO:0048857	Neural nucleus development	0.002744342	3
BP	GO:0009952	Anterior/posterior pattern specification	0.003091123	5
BP	GO:0048663	Neuron fate commitment	0.003139052	3
BP	GO:0021872	Forebrain generation of neurons	0.003277948	3
BP	GO:0009914	Hormone transport	0.003307998	6
BP	GO:0042108	Positive regulation of cytokine biosynthetic process	0.003420562	3
BP	GO:0051588	Regulation of neurotransmitter transport	0.003582686	4
BP	GO:0042535	Positive regulation of tumor necrosis factor biosynthetic process	0.003605656	2
BP	GO:0042742	Defense response to bacterium	0.003729911	6
BP	GO:0051966	Regulation of synaptic transmission, glutamatergic	0.003871012	3
CC	GO:0042734	Presynaptic membrane	7.9005E-05	6
CC	GO:0097060	Synaptic membrane	0.000641831	8
CC	GO:0098981	Cholinergic synapse	0.000846411	2

CC	GO:0098691	Dopaminergic synapse	0.001454534	2
CC	GO:0005892	Acetylcholine-gated channel complex	0.002507195	2
CC	GO:0035580	Specific granule lumen	0.002600802	3
CC	GO:0034774	Secretory granule lumen	0.00297311	6
CC	GO:0031045	Dense core granule	0.003134424	2
CC	GO:0060205	Cytoplasmic vesicle lumen	0.003828253	6
CC	GO:0031983	Vesicle lumen	0.003883687	6
CC	GO:0005766	Primary lysosome	0.004933391	4
CC	GO:0042582	Azurophil granule	0.004933391	4
CC	GO:0044305	Calyx of Held	0.004987225	2
CC	GO:0042581	Specific granule	0.00551604	4
CC	GO:0098793	Presynapse	0.005995437	7
CC	GO:0032809	Neuronal cell body membrane	0.006288323	2
CC	GO:0044298	Cell body membrane	0.006752734	2
CC	GO:0035578	Azurophil granule lumen	0.007623018	3
CC	GO:0034702	Ion channel complex	0.010711379	5
MF	GO:0004252	Serine-type endopeptidase activity	0.000120649	6
MF	GO:0008236	Serine-type peptidase activity	0.000243133	6
MF	GO:0017171	Serine hydrolase activity	0.000273314	6
MF	GO:0005179	Hormone activity	0.000302883	5
MF	GO:0048018	Receptor ligand activity	0.000503435	9
MF	GO:0004175	Endopeptidase activity	0.001022595	8
MF	GO:0015643	Toxic substance binding	0.001219163	2
MF	GO:0008201	Heparin binding	0.00132645	5
MF	GO:0022848	Acetylcholine-gated cation-selective channel activity	0.002618819	2

Abbreviations: DEGTGs: differentially expressed gefitinib target genes; GO: gene ontology; BP: biological process; MF: molecular function; CC: cell component; LUAD: lung adenocarcinoma.
